# Mindfulness-based therapy for insomnia for older adults with sleep difficulties: a randomized clinical trial

**DOI:** 10.1017/S0033291721002476

**Published:** 2023-02

**Authors:** Francesca Perini, Kian Foong Wong, Jia Lin, Zuriel Hassirim, Ju Lynn Ong, June Lo, Jason C. Ong, Kinjal Doshi, Julian Lim

**Affiliations:** 1 Centre for Sleep and Cognition, Yong Loo Lin School of Medicine, National University of Singapore, Singapore; 2 Feinberg School of Medicine, Northwestern University, Evanston, IL 60208, USA; 3 Department of Psychology, Singapore General Hospital, Singapore; 4 Department of Psychology, National University of Singapore, Singapore

**Keywords:** Mindfulness, Sleep disturbances, Sleep quality, Randomized clinical trial, Actigraphy, Polysomnography

## Abstract

**Objective:**

Poor sleep is a modifiable risk factor for multiple disorders. Frontline treatments (e.g. cognitive-behavioral therapy for insomnia) have limitations, prompting a search for alternative approaches. Here, we compare manualized Mindfulness-Based Therapy for Insomnia (MBTI) with a Sleep Hygiene, Education, and Exercise Program (SHEEP) in improving subjective and objective sleep outcomes in older adults.

**Methods:**

We conducted a single-site, parallel-arm trial, with blinded assessments collected at baseline, post-intervention and 6-months follow-up. We randomized 127 participants aged 50–80, with a Pittsburgh Sleep Quality Index (PSQI) score ⩾5, to either MBTI (*n* = 65) or SHEEP (*n* = 62), both 2 hr weekly group sessions lasting 8 weeks. Primary outcomes included PSQI and Insomnia Severity Index, and actigraphy- and polysomnography-measured sleep onset latency (SOL) and wake after sleep onset (WASO).

**Results:**

Intention-to-treat analysis showed reductions in insomnia severity in both groups [MBTI: Cohen's effect size *d* = −1.27, 95% confidence interval (CI) −1.61 to −0.89; SHEEP: *d* = −0.69, 95% CI −0.96 to −0.43], with significantly greater improvement in MBTI. Sleep quality improved equivalently in both groups (MBTI: *d* = −1.19; SHEEP: *d* = −1.02). No significant interaction effects were observed in objective sleep measures. However, only MBTI had reduced WASO_actigraphy_ (MBTI: *d* = −0.30; SHEEP: *d* = 0.02), SOL_actigraphy_ (MBTI: *d* = −0.25; SHEEP: *d* = −0.09), and WASO_PSG_ (MBTI: *d* = −0.26; SHEEP (*d* = −0.18). There was no change in SOL_PSG_. No participants withdrew because of adverse effects.

**Conclusions:**

MBTI is effective at improving subjective and objective sleep quality in older adults, and could be a valid alternative for persons who have failed or do not have access to standard frontline therapies.

## Introduction

Sleep problems are common in the general population (Léger, Poursain, Neubauer, & Uchiyama, 2008) with 25–50% of individuals reporting insufficient or non-restorative sleep. Due largely to morbidity, sleep quality tends to worsen with age (Lavoie, Zeidler, & Martin, 2018), contributing to further health risks, including cardiovascular disease (Cappuccio, Cooper, D'Elia, Strazzullo, & Miller, 2011), cognitive impairment (Bubu et al., 2017), and higher mortality (Cappuccio, D'Elia, Strazzullo, & Miller, 2010). Non-pharmacological interventions such as Cognitive-Behavioral Therapy for Insomnia (CBT-I) are effective at improving sleep quality, with medium effect sizes on insomnia severity and sleep efficiency (Trauer, Qian, Doyle, Rajaratnam, & Cunnington, 2015; van der Zweerde, Bisdounis, Kyle, Lancee, & van Straten, 2019) but have a non-response rate of up to 40% (Morin et al., 2009), motivating the investigation of treatment alternatives.

Mindfulness training is a core component of a ‘third wave’ of psychotherapies that are effective in treating a range of psychiatric disorders (Gross et al., 2011). As defined by Kabat-Zinn (1990), cultivating mindfulness involves paying attention to the present moment in a particular, intentional way, and bringing attitudes of acceptance and non-judgment to the experiences within it. Mindfulness is thought to enhance meta-awareness (Jankowski & Holas, 2014), which in turn allows for more flexible and adaptive responses to anxious or ruminative thoughts around sleep (Ong, Ulmer, & Manber, 2012). This contrasts with cognitive-behavioral approaches in that challenging dysfunctional thoughts and beliefs directly is not a primary goal of the treatment. Additionally, mindfulness training reduces chronic stress (Khoury, Sharma, Rush, & Fournier, 2015), and also specifically addresses the issue of pre-sleep arousal and other cognitive-emotional factors associated with insomnia (Ong et al., 2014; Ong, Xia, Smith-Mason, & Manber, 2018). This de-arousal may shorten sleep latency and consolidate sleep (Bonnet & Arand, 1997); moreover, pre-sleep arousal and sleep reactivity have been identified as potential predictors of future occurrence of insomnia (Kalmbach et al., 2018; Ong, Shapiro, & Manber, 2009).

Numerous studies have tested the effects of non-sleep-targeted mindfulness interventions on sleep quality, typically employing self-report measures such as the Pittsburgh Sleep Quality Index (PSQI) (Blake et al., 2016; Gross et al., 2011). Meta-analyses indicate a large effect of mindfulness training on improving such scores (Gong et al., 2016; Rusch et al., 2018). However, greater rigor in two areas is still needed. First, there is considerable heterogeneity across studies in the types of mindfulness training administered, including treatments that are not specifically targeted at sleep difficulties, making it challenging to compare results and provide specific treatment recommendations. Second, relatively few studies have reported the effects of mindfulness training on objective measures of sleep such as actigraphy or polysomnography [with exceptions (Britton, Haynes, Fridel, & Bootzin, 2010, 2012; Ong et al., 2014)], despite calls from both the mindfulness (Van Dam et al., 2017) and sleep (Montgomery, 2004) communities for such trials. Third, many studies using MBIs (Mindfulness-Based Interventions) did not use active control groups to control for attention and experimenter contact.

In this randomized trial, we attempted to address these three issues. We tested a newly manualized form of sleep-targeted mindfulness therapy, Mindfulness-Based Therapy for Insomnia [MBTI (Ong, 2017; Ong et al., 2012)], which combines techniques and exercises drawn from CBT-I with mindfulness exercises, inquiry, and home practice. A pilot study of MBTI on patients with chronic primary insomnia showed significantly greater symptom reduction compared with self-monitoring, and superior outcomes to a non-specific mindfulness-based program at 6-month follow-up (Ong et al., 2014). Here, we tested a larger group of older adults with more heterogeneous sleep difficulties studying both subjective and objective sleep changes in a sample adequately powered for this purpose. In order to show that the treatment may be effective for a wider range of complaints, we studied a broader sample of individuals with sleep difficulties using more liberal inclusion criteria (i.e. PSQI scores ⩾5 rather than a clinical diagnosis of insomnia disorder). Finally, we tested MBTI against an active sleep education and exercise control condition, matching for experimenter contact and home practice time: a feature lacking in many prior studies of mindfulness and sleep.

## Methods

### Trial design and randomization

The Mindfulness to Improve Sleep Trial was a parallel-arm, single-site, randomized controlled study testing MBTI (Ong, 2017) against a Sleep Hygiene, Education, and Exercise Program (SHEEP). Interventions were administered face to face in groups. Participants were allocated to groups once recruitment numbers were sufficient to begin a set of two classes. Six runs were conducted, with class sizes ranging from six to 15 participants (mean = 10.6, s.d. = 2.78).

After baseline measures were collected, participants were allocated to groups in a 1:1 ratio using computer-generated simple randomization of study identification numbers assigned at screening visit by author FP.

The authors assert that all procedures contributing to this study comply with the ethical standards of the relevant national and institutional committees on human experimentation and with the Helsinki Declaration of 1975, as revised in 2008. This trial was approved by the SingHealth Clinical Institution Review Board in November 2017 (identifier 2017-2830) and the Institutional Review Board of the National University of Singapore, and registered at the start of participants recruitment, but before randomization and the start of interventions. All participants provided written informed consent prior to enrolment.

### Participants

Participants were recruited through advertising in print media and online, through a local meditation center and sleep clinics and by word-of-mouth. Volunteers were pre-screened for the following eligibility criteria:

1. Age 50–80
2. Fluency in English
3. No cognitive impairment, defined as Mini-Mental State Examination (Folstein, Folstein, & McHugh, 1975) score ⩾26 and Montreal Cognitive Assessment (Nasreddine et al., 2005) score ⩾23
4. Self-reported sleep problems over the past month, defined as a PSQI (Buysse, Reynolds, Monk, Berman, & Kupfer, 1989) score ⩾5, AND at least one of the following sleep difficulties:
  - average reported sleep latency of >30 min
  - average wakefulness after sleep onset of >30 min
  - average total sleep time of <6.5 hr

We opted for a slightly more liberal cut-off point than the suggested PSQI >5 for poor sleep (Buysse et al., 1989); in practice, only four out of the 127 participants had a score of 5 at baseline.

Participants were excluded if they had major neurological disorders or psychiatric conditions (self-reported at screening, or reported suicidal ideation >1 in the Beck's Depression Inventory), contraindications for functional magnetic resonance imaging (fMRI) scanning (i.e. the presence of ferromagnetic objects or medical devices in the body; pregnancy), ongoing long-term use of sleep medications (assessed in the PSQI) or if they could not provide independent consent. Participants included in the study had never attended a mindfulness-based intervention.

Cognitive and questionnaire data were collected in the experimental suites of the Centre for Cognitive Neuroscience at Duke-NUS Medical School. Polysomnography was collected at participants' place of residence (see Appendix A in online Supplementary material for details). MBTI and SHEEP interventions were conducted in Duke-NUS Medical School or in a local wellness center. Participants were considered to have completed their intervention if they attended at least six out of the eight classes.

### Interventions

Appendix B contains the visit schedule and detailed syllabi of the two interventions, both containing eight weekly sessions of 2 hr each. Interventions were matched as closely as possible for contact time with instructors, amount of homework assigned, and for general content pertaining to sleep education.

The *MBTI* course was led by one of three certified mindfulness instructors with an average of ~800 hr of experience leading mindfulness-based courses, and ~2000 (1000–3000) hr of personal practice. Instructors were trained to administer the manualized version of MBTI by Ong (2017) with minor adjustments to suit the cultural context. Instructor adherence was maintained via an accompanying checklist for each class to ensure that all major learning points had been covered. All sessions after the first started with a formal mindfulness exercise (e.g. mindful eating, sitting meditation, mindful movement, and body scans). Descriptions of these practices can be found in Kabat-Zinn (1990). This was followed by a period of inquiry during which participants discussed their experiences during the past week, and the application of the practices and principles of mindfulness to their sleep difficulties. Most classes contained a didactic component pertaining to sleep disturbances (e.g. the etiology of insomnia, participants' relationship with sleep). The concept of sleep hygiene (Hauri, 1977) was introduced in Week 2, and behavioral strategies [sleep restriction therapy (Spielman, Saskin, & Thorpy, 1987) and stimulus control (Bootzin, Epstein, & Wood, 1991)] were introduced from Week 3. Participants were provided with a booklet with instructions on how to practice at home, and guided audio tracks (links given in Appendix B1). They were encouraged to practice for at least 20–30 min/day at the outset of the course, at least 6 days a week, and this time was increased incrementally to 45 min/day.

The *SHEEP* course was led by one of two instructors with at least a Master's degree in Clinical Psychology. SHEEP was developed by psychologists at the Singapore General Hospital as an active control condition, with matched contact and homework time. The course aimed to provide participants with information about sleep biology, self-monitoring of sleep behavior, and taught changes in habits and environment that could improve sleep quality (Appendix B2). In parallel with MBTI, each class contained a didactic component, a period of discussion, and a period where participants learned or practiced a sleep-promoting exercise. These exercises comprised diaphragmatic breathing, morning and evening stretching movements, and progressive muscle relaxation. Participants were provided with a weekly booklet containing instructions for home practice, and audio and video tracks. They were encouraged to practice every day with an equivalent total time schedule to the MBTI group.

### Outcomes

The primary self-reported outcomes for the trial were scores on the Insomnia Severity Index (ISI) and the PSQI. The ISI (Morin, Belleville, Bélanger, & Ivers, 2011) is a 7-item questionnaire that assesses the severity of daytime and night time insomnia symptoms. The PSQI (Buysse et al., 1989) assesses global sleep quality based on a range of self-reported symptoms and difficulties.

We computed sleep onset latency (SOL) and wake after sleep onset (WASO) as primary outcome variables when analyzing the actigraphy and polysomnography (PSG) data (S1 and S2 Appendices). Actigraphy was measured for a minimum of 5 days using a Phillips Actiwatch-2 or Actiwatch-Spectrum (Philips Respironics Inc., Pittsburgh, PA) worn on the non-dominant hand. PSG was collected using SOMNOtouch RESP recorders (SOMNOmedics GrmbH, Randersacker, Germany) in participants' homes. Previous studies have reported that ambulatory PSG data for insomnia do not show a first-night or reverse first-night effect, mitigating the need for a habituation night, while better reflecting a typical night of sleep for the subject (Bruyneel, Libert, Ameye, & Ninane, 2015; Herbst et al., 2010).

Secondary self-reported outcome measures included the Five Facet Mindfulness Questionnaire [FFMQ (Baer, Smith, Hopkins, Krietemeyer, & Toney, 2006)], Pre-Sleep Arousal Scale [PSAS (Nicassio, Mendlowitz, Fussell, & Petras, 1985)], and the Dysfunctional Beliefs and Attitudes about Sleep questionnaire [DBAS (Morin, 1993)]. Secondary objective measures included time-in-bed (TIB), total sleep time (TST) and sleep efficiency (SE).

All outcomes were assessed at pre and post intervention. PSQI and PSAS were additionally collected at the midpoint of the intervention and at 6-month follow-up via telephone.

### Blinding

Participants were not blind to condition, and were aware of the nature of both study interventions. However, all efforts were taken to present both MBTI and SHEEP as credible sleep treatments in recruitment material and during correspondence with participants.

Researchers assigned to collect questionnaire/PSG/MRI data from participants were blind to condition. PSG/actigraphy datasets were re-labeled and shuffled prior to scoring so that scorers were blind to group and time point.

### Sample size

The effects of mindfulness training on self-reported sleep variables are in the moderate [*d* = 0.46–0.61 (Gross et al., 2011)] to large [*d* = 0.89 (Black, O'Reilly, Olmstead, Breen, & Irwin, 2015)] range, and are large for actigraphic measures [*d* > 0.8 (Ong et al., 2014)]. Using a conservative value of *d* = 0.6 (*f* = 0.3) and with the study powered at *β* = 0.9, we determined that a sample size of *n* = 111 was needed to detect significant between-group effects at post-intervention at *p* = 0.05 (uncorrected, two-tailed, two measurements). We thus aimed to recruit 120 participants.

### Statistical methods

ITT analysis was conducted by filling in missing data with the pooled average of five imputed values derived from an iterative Markov Chain Monte Carlo method with predictive mean matching. Demographic and pre-intervention measures, including the State-Trait Anxiety Inventory [STAI (Spielberger, Gorsuch, Lushene, Vagg, & Jacobs, 1983], were used as predictor variables. Additional models were also run using the Beck Depression Inventory [BDI (Beck, Steer, & Brown, 1996)] as a predictor variable.

We analyzed primary and secondary variables of interest using 2 × 2 repeated measures analysis of variance, using Time (Pre/Post) as a within-subjects factor and Group (MBTI/SHEEP) as a between-subjects factor. We corrected for multiple comparisons of the six primary outcome variables using a Bonferroni threshold of *α* = 0.0125 (0.05/4) as we did not consider the actigraph and PSG-derived variables to be fully independent. Secondary comparisons were considered statistically significant at *α* = 0.05; these analyses should be considered exploratory. Effect sizes and confidence intervals were computed using estimation statistics, a non-parametric method that employs bootstrap resampling (Ho, Tumkaya, Aryal, Choi, & Claridge-Chang, 2019). Exploratory analysis on complete cases also tested the association between changes in self-reported trait mindfulness and subjective sleep quality using Pearson's correlation.

Statistical analysis was conducted with IBM SPSS Statistics for Windows Version 26 (IBM Corp., Armonk, NY).

## Results

### Participant flow

**Fig. 1.**
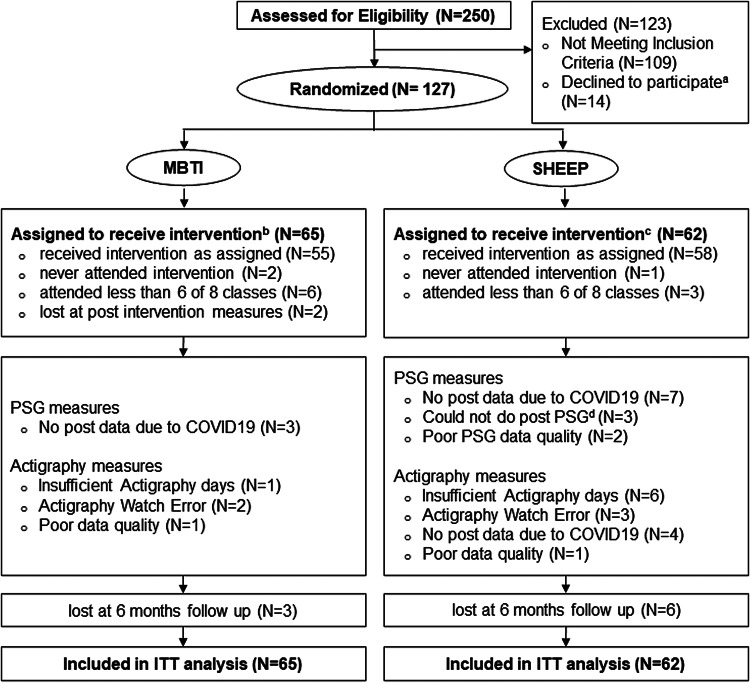
CONSORT chart showing the flow of participants through the trial. MBTI, mindfulness-based therapy for insomnia; SHEEP, sleep hygiene exercise and education program; PSG, polysomnography. (a) Reasons included time limitations (*n* = 2); concerned about MRI (*n* = 2) and lost interest in study (*n* = 10). b. Reasons for dropping out in MBTI included time limitations (*n* = 4), health reasons not related to sleep (*n* = 5) and unhappy with course content (*n* = 1); c. Reasons for dropping out in SHEEP included time limitations (*n* = 2) and unhappiness with course content (*n* = 2). d. One participant did not consent to do PSG at post-intervention, 2 participants could not do PSG due to sensitivity to electrode paste used.

Figure 1 shows the CONSORT diagram for this study. One hundred and twenty seven participants were randomized, of which 113 completed the intervention and provided at least one primary outcome measure. Participants attended on average 7.4 sessions (online Supplementary Fig. S1). Exit interviews indicated that few participants dropped out for study-related reasons (Fig. 1). Comparisons of demographics and baseline measures between dropouts and completers showed that dropouts were more likely to be male (*p* = 0.03), with no significant differences in clinical variables (online Supplementary Table S2).

Data were collected between August 2018 and October 2020. Recruitment stopped in February 2020 when we reached our predetermined target sample size of *N* = 120. However, due to restrictions put in place because of the COVID-19 pandemic, we were unable to collect actigraphy and PSG data from some participants (*N* = 11 and 4, respectively) in the final round of interventions.

No outliers were excluded from analysis. Groups did not significantly differ in baseline levels of any demographic characteristics (Table 1), or clinical characteristics (Table 2).

**Table 1.** Baseline demographic by intervention group and total sample

Characteristic	ALL^a^ (*N* = 127)		MBTI (*N* = 65)		SHEEP (*N* = 62)	
	Mean	s.d.	Mean	s.d.	Mean	s.d.
Age, mean (s.d.), years	60.9	6.4	61.2	6.6	60.7	6.2
	*N*	%	*N*	%	*N*	%
Female sex	74	58.3	36	55.4	38	61.3
Occupation						
Employed	47	37.0	24	36.9	23	37.1
Unemployed (retired, housewife)	59	46.5	29	44.6	30	48.4
Unknown	21	16.5	12	18.5	9	14.5
Race/ethnicity						
Chinese	120	94.5	63	96.9	57	91.9
Malay	0	0.0	0	0.0	0	0.0
Indian	2	1.6	1	1.5	1	1.6
Others	5	3.9	1	1.5	4	6.5
Education level						
Less than or equals primary	1	0.8	0	0.0	1	1.6
Less than or equals secondary	23	18.1	14	21.5	9	14.5
More than secondary or less than university	34	26.8	16	24.6	18	29.0
University	44	34.7	21	32.3	23	37.1
Post-graduate	25	19.7	14	21.5	11	17.7
Religion						
No religion	40	31.5	21	32.3	19	30.7
Buddhist	17	13.4	5	7.7	12	19.4
Hindu	3	2.4	2	3.1	1	1.6
Christian	52	40.9	28	43.1	24	38.7
Muslim	1	0.8	0	0.0	1	1.6
Others	7	5.5	4	6.2	3	4.8
Prefer not to say/unknown	7	5.5	5	7.7	2	3.2
Sleeping arrangement						
Sharing bed with partner	60	47.2	32	49.2	28	45.2
Not sharing bed	61	48.0	29	44.6	32	51.6
Unknown	6	4.7	4	6.2	2	3.2
Marital status						
Single	29	22.8	14	21.5	15	24.2
Married	80	63.0	42	64.6	38	61.3
Separated	2	1.6	1	1.5	1	1.6
Widowed	8	6.3	5	7.7	3	4.8
Divorced	7	5.5	2	3.1	5	8.1
Unknown	1	0.8	1	1.5	0	0.0

MBTI,  mindfulness based therapy for insomnia; SHEEP, sleep hygiene exercise, and education program; s.d., standard deviation.

a No significant differences by intervention arm for any variable.

**Table 2.** Intent-to-treat model estimates for primary and secondary outcome measures

Characteristic			MBTI (*N* = 65)								SHEEP (*N* = 62)								*p* value for baseline group comparison
			Pre		Week 4		Post		6 m FU		Pre		Week 4		Post		6 m FU		
			Mean	s.d.	Mean	s.d.	Mean	s.d.	Mean	s.d.	Mean	s.d.	Mean	s.d.	Mean	s.d.	Mean	s.d.	
		Primary outcomes																	
Survey	PSQI		10.98	3.10	9.14^b^	2.74	7.34^b^	3.01	7.74^b^	3.09	10.87	3.10	8.82^b^	3.50	7.58^b^	3.37	7.98^b^	3.28	0.84
	ISI		14.89	3.89	–		9.95^b^	3.88	–		14.21	4.13	–		11.23^b^	4.54	–		0.34
			(mins)		(mins)		(mins)		(mins)		(mins)		(mins)		(mins)		(mins)		
Actigraphy	WASO		74.70	37.89	–		64.87^b^	26.74	–		64.37	23.87	–		64.93	21.43	–		0.07
	SOL		22.89	27.39	–		17.46^a^	13.79	–		22.42	21.92	–		20.75	15.50	–		0.92
PSG	WASO		95.38	61.86	–		80.28^a^	55.04	–		71.72	40.24	–		64.66	40.62	–		0.01
	SOL		20.61	16.32	–		20.53	19.03	–		21.92	21.38	–		20.78	20.72	–		0.70
			Mean	s.d.	Mean	s.d.	Mean	s.d.	Mean	s.d.	Mean	s.d.	Mean	s.d.	Mean	s.d.	Mean	s.d.	
		Secondary outcomes																	
Survey	FFMQ		128.83	17.13	–		131.43	16.59	–		131.68	16.38	–		132.61	14.30	–		0.34
	PSAS Somatic		11.98	5.06	11.31	3.76	11.18	3.45	10.09^b^	2.82	11.69	4.00	11.47	3.88	11.11	3.49	10.46^a^	3.80	0.72
	PSAS Cognitive		19.51	5.40	17.91^a^	5.54	16.72^b^	4.48	15.71^b^	4.56	20.60	5.82	19.10^a^	6.08	18.29^b^	6.74	16.18^b^	6.46	0.28
	DBAS		4.82	1.14	–		4.06^b^	1.09	–		4.74	1.05	–		4.20^b^	1.07	–		0.70
			(mins)		(mins)		(mins)		(mins)		(mins)		(mins)		(mins)		(mins)		
Actigraphy	TST min		364.41	57.70	–		361.38	54.50	–		365.45	56.47	–		360.71	44.58	–		0.92
	TIB min		462.01	64.33	–		443.71^b^	51.28	–		452.25	48.43	–		446.54	40.47	–		0.34
	SE %		79.40	10.83	–		81.43^a^	8.22	–		80.52	8.22	–		80.53	6.30	–		0.51
PSG	TST min		348.06	71.48	–		373.19^b^	61.65	–		343.67	67.72	–		375.50^b^	71.07	–		0.72
	TIB min		442.68	74.39	–		455.19	70.05	–		460.27	68.46	–		470.75	69.50	–		0.17
	SE %		78.69	10.21	–		82.29^a^	7.89	–		75.08	13.00	–		80.04^b^	11.07	–		0.09

MBTI, mindfulness-based therapy for insomnia; SHEEP, sleep hygiene exercise and education program; SD, standard deviation; PSQI, Pittsburg's sleep quality index; min, minutes; ISI, insomnia symptoms index; WASO, wake after sleep onset; PSG, polysomnography; SOL, sleep onset latency; FFMQ, Five Facets Mindfulness Questionnaire; PSAS, Pre Sleep Arousal Scale; DBAS, dysfunctional beliefs about sleep; TST, total sleep time; TIB, total time in bed; SE, sleep efficiency.

^a^Significant change from baseline at *p* < 0.05, uncorrected.

^b^Significant change from baseline at *p* < 0.01, uncorrected.

### Analysis of primary outcomes

Table 2 and online Supplementary Table S1 summarize the ITT model estimates and inferential statistics for all primary and secondary analyses.

**Fig. 2.**
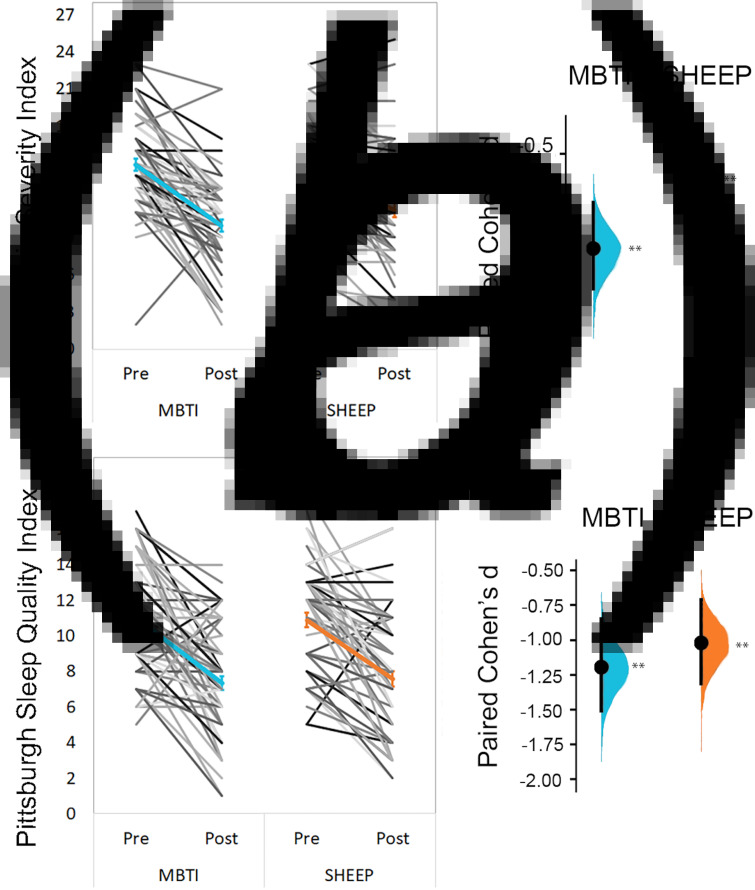
Primary self-reported outcomes**.** Left graphs in each panel depict mean change (and standard error) from pre- to post-intervention, and trajectory of change for individual participants. Right graphs show the distribution for effect size of change drawn from 5000 bootstrap samples. (a) Insomnia severity is reduced in both study groups, with significantly greater reductions in MBTI (MBTI: *d* = −1.27, 95% CI −1.61, −0.89; SHEEP: *d* = −0.69, 95% CI −0.96, −0.43). (b) Self-reported sleep quality increases in both groups, with no significant difference between groups (MBTI: *d* = −1.19, 95% CI −1.51, −0.85; SHEEP: *d* = −1.02, 95% CI −1.31, −0.71). MBTI = Mindfulness Based Therapy for Insomnia; SHEEP = Sleep Hygiene Exercise, and Education program. **p < 0.01.

On the ISI, we found a significant time × group interaction [*F*_(125,1)_ = 6.89, *p* = 0.010], with MBTI showing a significantly greater reduction in insomnia severity than SHEEP. Estimation analysis confirmed that both groups improved from baseline [MBTI: *d* = −1.27, 95% confidence interval (CI) −1.61 to −0.89; SHEEP: *d* = −0.69, 95% CI −0.96 to −0.43] (Fig. 2a).

Both groups reported improved sleep quality scores over time with no significant time × group interactions on the PSQI (Fig. 2b). Bootstrap analysis showed significant improvement at week 4 (MBTI: *d* = −0.63, 95% CI −0.90 to −0.36); SHEEP: *d* = −0.62, 95% CI −0.90 to −0.36), and further change in sleep quality post-intervention in both MBTI (*d* = −1.19, 95% CI −1.51 to −0.85) and SHEEP (*d* = −1.02, (95% CI −1.31 to −0.71). Improvement was also maintained at the 6-month follow-up (MBTI: *d* = −1.05, 95% CI −1.37 to −0.73; SHEEP: *d* = −0.91, 95% CI −1.21 to −0.63), with no change from post-intervention.

**Fig. 3.**
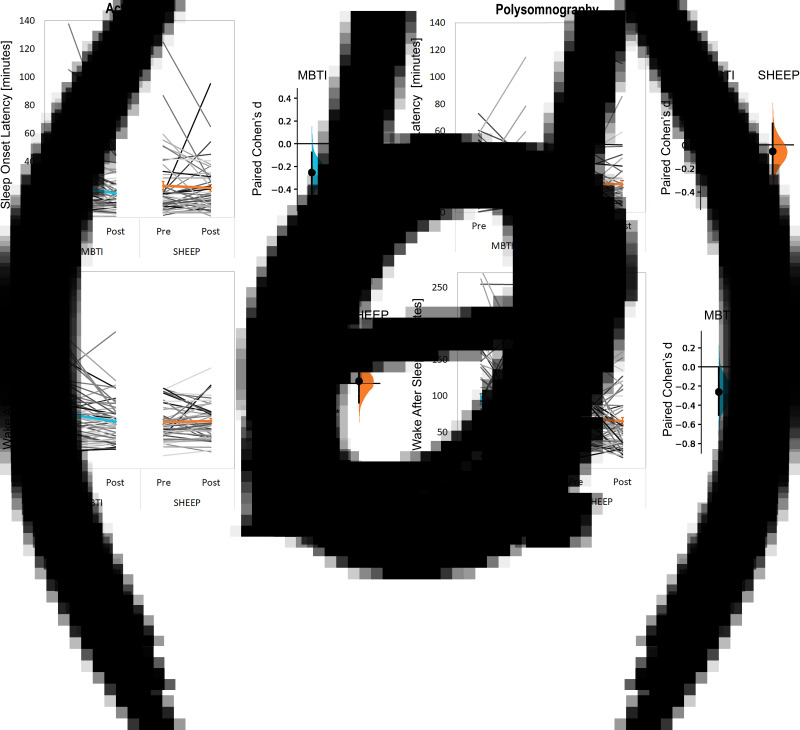
Primary objective measures. Left graphs in each panel depict mean change (and standard error) from pre- to post-intervention, and trajectory of change for individual participants. Right graphs show the distribution for effect size of change drawn from 5000 bootstrap samples. (a, c) Actigraphy measures. Sleep onset latency (MBTI: *d* = −0.25, 95% CI −0.45, −0.08; SHEEP: *d* = −0.09, 95% CI −0.32 0.21) and wake after sleep onset (MBTI: *d* = −0.30, 95% CI −0.49, −0.10; SHEEP: *d* = 0.02, 95% CI −0.21, 0.27) were reduced in MBTI but not SHEEP. (b, d) PSG measures. No significant change was observed in either treatment group. MBTI = Mindfulness Based Therapy for Insomnia; SHEEP = Sleep Hygiene Exercise, and Education program. *p < 0.05; **p < 0.01.

The time × group interaction in SOL_actigraphy_ (time taken to fall asleep) was not significant. However, bootstrap analysis showed reduced SOL_actigraphy_ in MBTI (*d* = −0.25, 95% CI −0.45 to −0.077), while SOL_actigraphy_ changes in SHEEP were not significant (*d* = −0.088, 95% CI -0.32 to 0.21) (Fig. 3a). The time × group interaction in WASO_actigraphy_ was significant [*F*(125,1), = 5.68; *p* = 0.019], with MBTI participants showing a greater decrease in time spent awake at night compared to SHEEP, but did not survive correction for multiple comparisons. However, bootstrap analysis showed a significant reduction in WASO_actigraphy_ in MBTI (*d* = −0.30, 95% CI −0.49 to −0.10), but not SHEEP (*d* = 0.025, 95% CI −0.21 to 0.27) (Fig. 3c).

We found no significant interaction for the polysomnographic primary outcomes SOL_PSG_ and WASO_PSG_. Bootstrap analysis showed no change in SOL_PSG_ in either treatment group (MBTI: *d* = −0.0043, 95% CI −0.27 to 0.25; SHEEP: *d* = −0.054, 95% CI −0.25 to 0.18) (Fig. 3b). MBTI had significantly decreased WASO_PSG_ (*d* = −0.26, 95% CI −0.50 to −0.0057), while SHEEP had no change (*d* = −0.18, 95% CI −0.47 to 0.080) (Fig. 3d).

### Analysis of secondary outcomes

We found no significant time × group interaction in self-reported mindfulness. Neither group increased significantly in mean (s.d.) mindfulness in bootstrap analysis (MBTI: *d* = 0.15, 95% CI −0.015 to 0.33; SHEEP: *d* = 0.061, 95% CI −0.12 to 0.24).

We found no time × group interaction on the PSAS in either somatic or cognitive arousal. Somatic arousal did not decrease significantly in either group at Week 4 (MBTI: *d* = −0.15, 95% CI −0.41 to 0.13; SHEEP: *d* = −0.057, 95% CI −0.27 to 0.18) or at post intervention (MBTI: *d* = −0.19, 95% CI −0.45 to 0.084; SHEEP: *d* = −0.16, 95% CI −0.40 to 0.11), but showed a significant decrease at 6-month follow-up (MBTI: *d* = −0.46, 95% CI −0.70 to −0.15; SHEEP: *d* = −0.32, 95% CI −0.60 to 0.013).

In contrast, cognitive arousal decreased significantly at Week 4 (MBTI: *d* = −0.29, (95% CI −0.58 to −0.023; SHEEP: *d* = −0.25, 95% CI −0.46 to −0.064), and at post intervention for both groups (MBTI: *d* = −0.56, 95% CI -0.87 to −0.30; SHEEP: *d* = −0.37, 95% CI −0.66 to −0.089), and again at follow-up (MBTI: *d* = −0.76, 95% CI −1.11 to −0.45; SHEEP: *d* = −0.72, 95% CI −1.04 to −0.41).

There was no time × group interaction in change in dysfunctional beliefs about sleep. Both groups had significantly lower DBAS scores post-intervention (MBTI: *d* = −0.68, 95% CI −0.92 to −0.44; SHEEP: *d* = −0.51, 95% CI −0.73 to −0.28).

In secondary actigraphy variables (TIB, TST, and SE), none of the interactions were significant. There was also no significant change in TST_actigraphy_ in either group (MBTI: *d* = −0.05, 95% CI −0.24 to 0.14; SHEEP: *d* = −0.093, 95% CI −0.33 to 0.14). A significant improvement in SE_actigraphy_ in the MBTI group was accompanied by a significant reduction in TIB_actigraphy_ (SE_actigraphy_: *d* = 0.21, 95% CI 0.031 to 0.42; TIB_actigraphy_: *d* = −0.32, 95% CI −0.55 to −0.11). There were no changes in either of these variables in the SHEEP group (SE_actigraphy_: *d* = 0.002, 95% CI −0.23 to 0.24); TIB_actigraphy_: *d* = −0.13, 95% CI −0.38 to 0.098).

In secondary PSG variables, none of the time × group interactions were significant. There was no significant change in TIB_PSG_ in either group (MBTI: *d* = 0.17, 95% CI −0.071 to 0.41), SHEEP: *d* = 0.15, 95% CI −0.11 to 0.43), but both groups had significantly increased TST_PSG_ [MBTI: from 348.06 (71.48) at baseline to 373.19 (61.65) post-intervention, *d* = 0.38, 95% CI 0.11 to 0.62; SHEEP: from 343.67 (67.72) to 375.50 (71.07), *d* = 0.46, 95% CI 0.11 to 0.79]. Both groups also had significantly improved SE_PSG_ (MBTI: *d* = 0.39, 95% CI 0.088 to 0.69; SHEEP: *d* = 0.41, 95% CI 0.12 to 0.68).

Based on self-report, we found no evidence that ambulatory PSG affected participants' sleep relative to usual (Appendix A3).

### Other exploratory analyses

We tested for sex differences using an ITT model including sex as a covariate for all primary and secondary outcomes. Results are reported in online Supplementary Table S4. Sex did not show a significant interaction with time for any of the outcomes (all *p* values >0.12), and the results did not change materially from the repeated measures ANOVA (rmANOVA) results without covariates.

Two additional ITT models were run with depression scores at baseline as a predictor variable in addition to the other predictors used in the main analysis. Results of the analysis of variance (ANOVA) using these imputed datasets are presented in online Supplementary Table S5. Using these models, we found a significant time × group interaction in FFMQ (BDI only: *p* = 0.018; BDI and STAI: *p* = 0.007), driven by increases in MBTI. All other results remained materially unchanged (comparisons with main ITT analysis in online Supplementary Table S5).

To explore if different mechanisms may be involved in the two interventions, we correlated change in self-reported mindfulness with change in ISI and PSQI. These associations were significant in MBTI (PSQI: *r* = −0.59, ISI: *r* = −0.38, *p* values <0.01), but not SHEEP (PSQI: *r* = 0.01, *p* = .97; ISI: *r* = 0.09, *p* = 0.52), and correlation values differed significantly between the groups (PSQI: *z* = −3.52; ISI: *z* = −2.49; *p* values <0.01; online Supplementary Fig. S2).

Treatment acceptability and satisfaction about course content and instructors were assessed at the conclusion of the course. These measures were not significantly different between groups (Appendix D).

### Adverse effects

No adverse events related to the interventions and study visits were reported.

## Discussion

MBTI is a relatively novel program that comprises empirically supported behavioral strategies (e.g. sleep restriction and stimulus control) with mindfulness instruction, inquiry, and practice. The current trial is the first to compare manualized MBTI against an active control condition in an adequately powered sample, and represents an advance along the logical progression of studies of this intervention. In line with previous study, MBTI had a large effect in improving self-reported sleep quality and reducing insomnia symptoms. While there was no difference in PSQI improvement between MBTI and the control condition, this was likely due to the large change in SHEEP compared with effects observed in prior studies of sleep hygiene programs (Chung et al., 2018). A likely explanation for this difference was that SHEEP contained many active components (e.g. exercise, breathing training, and relaxation) compared with typical sleep hygiene courses. In contrast, we observed that MBTI was superior to SHEEP in reducing insomnia severity, measured using the ISI. A key difference between the ISI and the PSQI is that the former scale gives more weight to daytime impairment. Given the large and equal improvements in PSQI scores between the groups, the result on the ISI suggests that MBTI participants may have been more accepting of sensations of sleepiness or fatigue on nights with poor-quality sleep.

Consistent with the first report on MBTI, we also observed significant, albeit smaller effects in actigraphic sleep measures and on single-night PSG, with benefits seen in total sleep time and sleep efficiency. These data provide confirmatory evidence that manualized MBTI is efficacious in improving some aspects of objective sleep duration, which is in turn a potentially important predictor of cognitive decline (Djonlagic et al., 2020) and mortality associated with cardiometabolic factors (Fernandez-Mendoza, He, Vgontzas, Liao, & Bixler, 2019).

In summary, of our six primary comparisons, only one (ISI) showed a significant time × group interaction. Differences in objective measures were seen in estimation tests but not ANOVA, where effect sizes were significant for three out of four variables in the MBTI and zero out of four in SHEEP. Furthermore, none of the changes in secondary variables differed between groups. The incremental benefit of MBTI over active control was, as such, limited to only a few outcomes, with insomnia severity being the most notable. Intervention effects did not differ between male and female participants. Overall, these findings are in line with the current literature of MBIs on sleep (Ong & Moore, 2020).

We did not observe a significant increase in mindfulness scores in either group, possibly because of a ceiling effect (baseline mindfulness scores tend to be high in older individuals, as this variable is correlated with age). We also note that many studies with mindfulness interventions also do not find increases in self-reported mindfulness (Visted, Vøllestad, Nielsen, & Nielsen, 2014), suggesting a need for improved ways to measure this construct (Grossman, 2011). Notwithstanding these issues, exploratory analysis of our data showed that sleep quality improvement and insomnia score decreases were associated with increases in self-reported trait mindfulness in the MBTI group only, suggesting that the mechanism of change differed between the groups, and that cultivation of this ability may be an important ingredient in symptom reduction.

Although we did not compare directly MBTI with a CBT-I, the current frontline treatment for primary insomnia, our trial showed that the effect size of the reduction in ISI scores is comparable to those seen in studies of CBT-I (van Straten et al., 2018). MBTI also reduces pre-sleep arousal, which is a predictor of non-remission/relapse of future episodes of insomnia, and addresses a specific shortcoming of CBT-I (Kalmbach et al., 2018; Ong et al., 2009). Nevertheless, further studies are necessary to determine if MBTI has a role in clinical practice, for example a non-inferiority trial against CBT-I, or a trial recruiting patients who have failed this treatment. Our study also demonstrates that MBTI was effective when delivered in groups of up to 15 people, indicating that MBTI can be efficiently disseminated in a community-based program as opposed to CBT-I, which is delivered in clinics.

We conclude that MBTI may be an attractive alternative for those who did not respond or do not have access to CBT-I. Future studies should examine whether MBTI leads to changes in cognitive or neurological functioning, and probe more deeply into treatment mediators and moderators.

### Generalizability

The demographics of our study population differed considerably from the first report of MBTI (Ong et al., 2014), being older and primarily Han Chinese, and our inclusion criteria were much broader than the first study, which recruited only patients with primary insomnia and no comorbid disorders. This is the first evidence that the effects of MBTI are somewhat generalizable across age and culture and accrue to patients without a specific sleep diagnosis. However, because of the nature of recruitment (i.e. through advertisements), our sample was not representative of the general population in Singapore. We also studied older adults due to an interest in understanding the role of improving sleep in mitigating cognitive decline, potentially limiting generalizability of the findings to younger people.

### Strengths and limitations of study

This study represents an attention-controlled, adequately powered RCT testing a mindfulness-based intervention specifically targeting sleep difficulties, on both subjective and objective measures, including home polysomnography. The comparison with a highly active control intervention that is commonly used as an initial recommendation for people with insomnia and the low rate of dropouts are also strengths of the trial. One limitation of the study is that we elected not to take audio or video recordings of the interventions as we were concerned that these would interfere with participant's willingness to engage in the classes and share personal experiences. Because of this, we could not perform an unbiased assessment of instructor fidelity in implementing the protocol. Longer-term follow-up of both subjective and objective measures at different time intervals is also desirable in future research, possibly using tools such as commercial wearables or apps that allow for more widespread and extensive tracking.

## Data Availability

The study used identifiable individual patient data which will be subject to ethics, consent and privacy restrictions; however within these constraints we will make fully anonymized data available on request wherever possible. De-identified data from this trial will be shared upon request from the corresponding author Julian Lim.

## Supplementary material

For supplementary material accompanying this paper visit https://doi.org/10.1017/S0033291721002476. click here to view supplementary material
