# Supplementary material for: Mindfulness-based therapy for insomnia for older adults with sleep difficulties: a randomized clinical trial
Source: Psychol Med. 2021 Jul 1;53(3):1038–48. doi: 10.1017/S0033291721002476 (PMC9975962; doi:10.1017/S0033291721002476)
Supplement: Supplementary file 1 [file S0033291721002476sup001.zip › S0033291721002476sup001.docx]

**Appendix A: Polysomnography scoring protocol and supplementary results**

A.1 *Methods*

Polysomnography was recorded at participants’ homes using the portable SOMNOtouch RESP (SOMNOmedics GmbH, Randersacker,Germany). We used a 10-channel montage (M1, M2, C3, C4, Cz, Fpz, EOG-Left, EOG-Right, 2 Chin electrodes) following the international 10-20 system, which consisted of C3 and C4 referenced to the M1 and M2 mastoid channels, with online reference at Cz and ground at Fpz. Bilateral diagonally placed electrooculography (EOG) and bipolar submental electromyography (EMG) on the chin were also used. Data were recorded at 250 Hz sampling rate and filtered between 0.1 and 30 Hz.

Two members of staff arranged to apply the electrodes at the participant’s home at least 60 minutes before their habitual sleep time. Before leaving for the night, impedance was checked to be below 5kΩ. Participants were assured that they need not adjust their typical sleep habits in any particular way (e.g. making a particular effort to keep still). Participants were provided with a digital clock that was synchronised to the time on the SOMNOtouch RESP. and were instructed to record the time they went to sleep and woke up based on this clock. They were also asked to rate their sleep quality on several visual analog scales on awakening, specifically: the quality of their sleep, how refreshed they felt, and their sleep quality relative to their usual night of sleep.

PSG data were extracted using DOMINO light software (version 14.0; SOMNOmedics, GMBJm Randersacker, Germany) and exported to be scored using FASST in Matlab. In the event that PSG data quality was deemed unacceptable, we performed a second night of sleep recording whenever this was feasible.

Sleep recordings were scored by two trained technicians. Each technician staged 2/3s of all available nights with 1/3 of the nights double-scored to calculate inter-rater reliability. The average of the sleep variables which have been double-scored are reported along with the individually scored nights.

Bilateral electrooculography (EOG), bipolar submental electromyography (EMG) and central cross hemispheric re-referenced central electrodes (C3-A2, C4-A1) were used to assess sleep stages according to the AASM Manual for the Scoring of Sleep and Associated Events: Rules, Terminology and Technical Specifications for consistency. Time in bed (TIB) was calculated according to the wake and bed time specified by the participants, Sleep onset latency (SOL) was determined by the first non-wake and non N1 sleep epoch, total sleep time (TST) was calculated by the summation of all epochs scored between SOL and wake time excluding WASO.

*A.2 Supplementary results*

Inter-rater reliability in sleep scoring is summarized in Supplementary Table S2.

*A.3 Results from bedside questionnaire*

On the morning following PSG recording participants rated their sleep quality on an in-house Subjective Sleep Score Visual Analogue Scale (SSSVAS) consisting of three questions: 1) “*How was your sleep quality last night?*”; 2) “*Compared to your usual night of sleep, how would you rate the quality of sleep last night?* 3) “*How refreshed do you feel now?*” Participants did not report having worse-than-usual sleep on the PSG night (one-tailed t-test; pre-intervention: t = -1.637, p = 0.053; post-intervention: t = .974, p = 0.66). On the whole, rmANOVA showed that participants reported significantly better sleep quality on the post-intervention SSSVAS compared with pre-intervention, but estimations statistics showed that this was significant only in MBTI (d=0.49, 95%CI 0.16 to 0.85) and not in SHEEP (d=.23, 95%CI -0.16 to 0.65). Both groups reported that they were feeling more refreshed in the morning after the PSG post intervention compared to pre (MBTI d=0.68, 95%CI 0.34 to 1.04; SHEEP d=0.41, 95%CI 0.07 to 0.76). MBTI also reported better sleep compared to usual at postintervention (d=0.42, 95%CI 0.10 to 0.74), but not SHEEP participants (d=0.06, 95%CI -0.31 to 0.47). No significant time x group interactions were found in any of the variables (p values >0.14).
