# Supplementary material for: Mindfulness-based therapy for insomnia for older adults with sleep difficulties: a randomized clinical trial
Source: Psychol Med. 2021 Jul 1;53(3):1038–48. doi: 10.1017/S0033291721002476 (PMC9975962; doi:10.1017/S0033291721002476)
Supplement: Supplementary file 1 [file S0033291721002476sup001.zip › S0033291721002476sup002.docx]

**Appendix C: Actigraphy scoring protocol and supplementary results**

C.1 *Methods*

Philips Actiwatch 2 or Philips Actiwatch Spectrums (Philips Respironics Inc., Pittsburgh, PA) were employed to record 30- second epochs for at least 3 weekdays and 1 weekend of activity and white light levels before and after the intervention, including the nights of polysomnography recording. The same watch model was used for pre- and post- intervention recording. Participants were instructed to wear the actigraph on their non-dominant hand and to keep it on at all times except when engaging in sports and water-based activity. Sleep and wake times were also marked on the data by a button press on the watch.

A sleep diary was also issued for the period of the actigraphic recording. Participants were instructed to record their sleep time each night and in their wake time, estimated sleep onset latency, and wake after sleep onset the following morning.

All sleep and wake times on the actigraph were marked by two experienced technicians using the sleep diary as a guide. Scorers were blind to the group assignment of participants, and whether the recording had been taken pre- or post-intervention. An average of the sleep and wake time were taken if difference in the marked times between the technicians were less than 50 minutes. If the discrepancy was larger than 50 minutes, the author J.L., blind to the source, re-evaluated the data and resolved the discrepancy.

Sleep variables were calculated automatically using Actiware software (version 6.0.2) on a medium setting with a 10-minute sleep and wake window. Weekday and weekend sleep variables were weighted to a ratio of 5:2.
