# Supplementary material for: Mindfulness-based therapy for insomnia for older adults with sleep difficulties: a randomized clinical trial
Source: Psychol Med. 2021 Jul 1;53(3):1038–48. doi: 10.1017/S0033291721002476 (PMC9975962; doi:10.1017/S0033291721002476)
Supplement: Supplementary file 1 [file S0033291721002476sup001.zip › S0033291721002476sup003.docx]

**Appendix D: Treatment acceptability**

At the end of the intervention participants filled in a Satisfaction Survey adapted from The Satisfaction with Therapy and Therapist Scale (STTS-R) (Oei and Green 2008). Statements are scored on a scale of 1 to 5 where 1 represents “strongly disagree” and 5 represents “strongly agree”.

STTS-R was revised for our study to fit the type of interventions offered (e.g. “therapist” was replaced with “teacher”). We also added five questions to account for motivation and practice learned during the interventions (es: “I felt motivated to set time aside to use/practice the course material at home.”; “I practiced what learned in class most days.”) In addition, we also asked about participants’ satisfaction with initial allocation (“Prior to the start of the workshop, I was happy with my allocation to the group intervention.”). Thus, we divided items in three categories and averaged scores: Intervention (14 items); Teacher (4 items) and Randomization (1 item). 50 MBTI and 56 SHEEP participants completed the Satisfaction Survey. SHEEP participants were not as satisfied as MBTI regarding the initial allocation to intervention (MBTI: mean=4.06, SD=.80; SHEEP: mean=3.46, SD=.95; p<.01). However, ultimately both groups were highly satisfied with both intervention content (MBTI: mean=4.29, SD=.46; SHEEP: mean=4.19, SD=.41;) and teacher (MBTI: mean=4.47, SD=.45; SHEEP: mean=4.41, SD=.51), with no significant difference between treatments.

Reference:

Oei, T.P. S., & Green, A.L. (2008). The Satisfaction With Therapy and Therapist Scale-Revised (STTS-R) for group psychotherapy: Psychometric properties and confirmatory factor analysis. Professional Psychology: Research and Practice, 39 (4), 435-442. doi: 10.1037/0735-7028.39.4.435
