# Supplementary material for: Mindfulness-based therapy for insomnia for older adults with sleep difficulties: a randomized clinical trial
Source: Psychol Med. 2021 Jul 1;53(3):1038–48. doi: 10.1017/S0033291721002476 (PMC9975962; doi:10.1017/S0033291721002476)
Supplement: Supplementary file 1 [file S0033291721002476sup001.zip › S0033291721002476sup004.docx]

Supplement Figure 1 for Perini et al Mindfulness-Based Therapy for Insomnia for older adults with sleep difficulties: a randomized clinical trial.


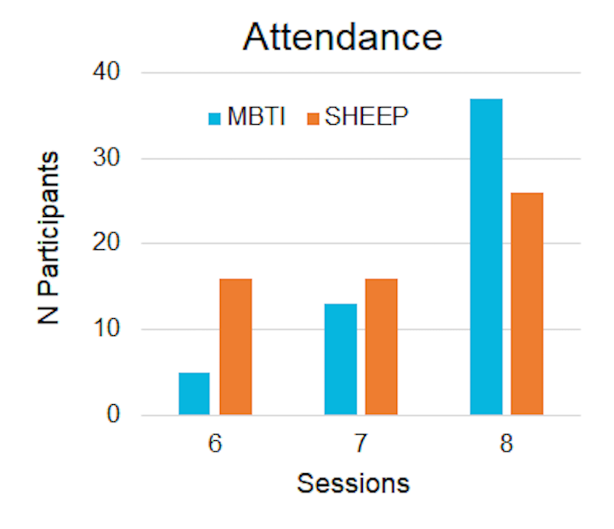


**Figure S1: Number of sessions attended by completers (N=113), including both group sessions and make-up sessions.** Participants needed to attend at least 6 classes out of 8 to be included in the study. They were offered the possibility to do a make up class with the teacher if they missed a class, for a maximum to 3 non-consecutive sessions. MBTI attendance was higher (mean=7.6; SD=.65) compared to SHEEP (mean=7.2; SD=.84); T(107.2)=2.89, p=.03), with no difference in make up classes attended (p=.08). MBTI = Mindfulness Based Therapy for Insomnia; SHEEP = Sleep Hygiene Exercise, and Education program.
