# Supplementary material for: Mindfulness-based therapy for insomnia for older adults with sleep difficulties: a randomized clinical trial
Source: Psychol Med. 2021 Jul 1;53(3):1038–48. doi: 10.1017/S0033291721002476 (PMC9975962; doi:10.1017/S0033291721002476)
Supplement: Supplementary file 1 [file S0033291721002476sup001.zip › S0033291721002476sup005.docx]

Supplement Figure 2 for Perini et al Mindfulness-Based Therapy for Insomnia for older adults with sleep difficulties: a randomized clinical trial.


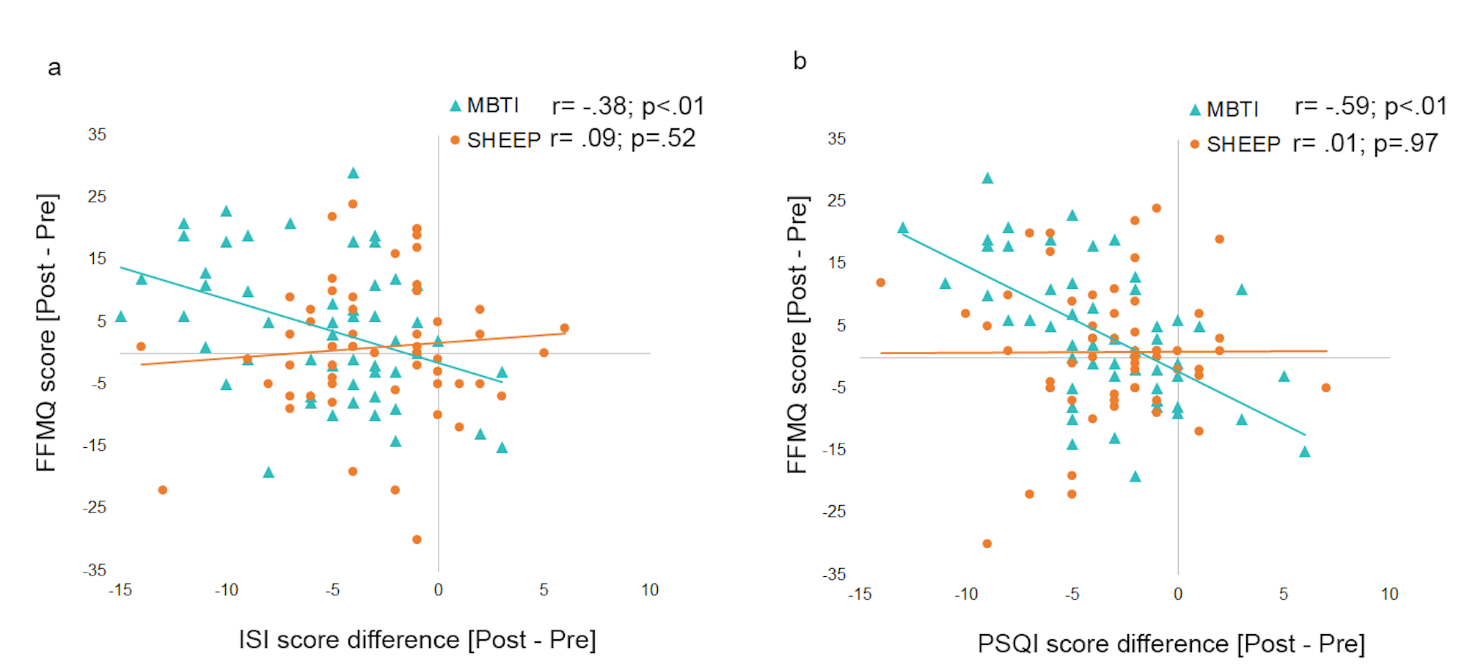


**Figure S2: Self-reported mindfulness correlates with changes in insomnia severity and sleep quality in the MBTI group only.** Graphs show association between changes in Mindfulness scores and Insomnia (a.), and changes in Mindfulness and Subjective Sleep Quality (b.), with MBTI participants in blue and SHEEP participants in orange. An increase in Mindfulness was associated with both a decrease in Insomnia and an increase in Sleep Quality, but only for MBTI group, as tested with Pearson’s correlation. MBTI = Mindfulness Based Therapy for Insomnia; SHEEP = Sleep Hygiene Exercise, and Education program.; FFMQ = Five Facet Mindfulness Questionnaire; ISI = Insomnia Severity Index; PSQI = Pittsburgh Sleep Quality Index.
