# Supplementary material for: Mindfulness-based therapy for insomnia for older adults with sleep difficulties: a randomized clinical trial
Source: Psychol Med. 2021 Jul 1;53(3):1038–48. doi: 10.1017/S0033291721002476 (PMC9975962; doi:10.1017/S0033291721002476)
Supplement: Supplementary file 1 [file S0033291721002476sup001.zip › S0033291721002476sup006.docx]

Supplement table 1 for Perini et al Mindfulness-Based Therapy for Insomnia for older adults with sleep difficulties: a randomized clinical trial

| **Table 1. Repeated Measures ANOVA F values for ITT analysis and complete-case analysis** | | | | | | | | | | | | | | | | | |
| --- | --- | --- | --- | --- | --- | --- | --- | --- | --- | --- | --- | --- | --- | --- | --- | --- | --- |
|  | **Primary Measures** | | | | | | **Secondary Measures** | | | | | | | | | | |
|  | **PSQI** | **ISI** | **PSG** | | **Actigraphy** | | **FFMQ** | **PSAS** | | **DBAS** | **PSG** | | | **Actigraphy** | | |  |
|  |  |  | WASO | SOL | WASO | SOL |  | Somatic | Cognitive |  | TST | TIB | SE | TST | TIB | SE |  |
| **Complete-case Analysis** | | | | | | | | | | | | | | | | | |
| Time | 96.28^a^ | 122.09^a^ | 6.99^b^ | 0.99 | 3.65 | 2.51 | 5.46^b^ | 2.62 | 23.23 ^a^ | 53.46^a^ | 5.27^b^ | 0.02 | 10.6^a^ | .03 | 4.39^b^ | 2.97 |  |
| Time*Group | 0.52 | 10.06^a^ | 0.06 | 0.04 | 5.47^b^ | 0.55 | 2.1 | 0.01 | 0.78 | 1.61 | 0.02 | 0.33 | 0.001 | .08 | 2.36 | 2.76 |  |
| **ITT Analysis** | | | | | | | | | | | | | | | | | |
| Time | 118.57^a^ | 113.12^a^ | 5.70^b^ | 0.15 | 4.53^b^ | 3.71 | 3.18 | 2.99 | 23.69^a^ | 60.93^a^ | 16.28^a^ | 3.27 | 17.43^a^ | 0.95 | 8.92^a^ | 2.49 |  |
| Time*Group | 0.31 | 6.89^a^ | 0.75 | 0.12 | 5.68^a^ | 1.04 | 0.71 | 0.08 | 0.21 | 1.66 | 0.23 | 0.03 | 0.44 | 0.05 | 2.46 | 2.43 |  |
| Abbreviations: MBTI, Mindfulness Based Therapy for Insomnia; SHEEP, Sleep Hygiene Exercise and Education program; SD, Standard Deviation; PSQI, Pittsburg’s Sleep Quality Index; ISI, Insomnia Symptoms Index; WASO, Wake After Sleep Onset; PSG, Polysomnography; SOL, Sleep Onset Latency; FFMQ, Five Facets Mindfulness Questionnaire; PSAS, Pre Sleep Arousal Scale; DBAS, Dysfunctional Beliefs about Sleep; TST, Total Sleep Time; TIB, total Time in Bed; SE, Sleep Efficiency. a = p value <0.01; b = p value <0.05; | | | | | | | | | | | | | | | | | |
