# Supplementary material for: Mindfulness-based therapy for insomnia for older adults with sleep difficulties: a randomized clinical trial
Source: Psychol Med. 2021 Jul 1;53(3):1038–48. doi: 10.1017/S0033291721002476 (PMC9975962; doi:10.1017/S0033291721002476)
Supplement: Supplementary file 1 [file S0033291721002476sup001.zip › S0033291721002476sup007.docx]

Data Table 2 for Perini et al Mindfulness-Based Therapy for Insomnia for older adults with sleep difficulties: a randomized clinical trial

| **Table 2 \| Comparison of characteristics between dropouts and completers (mean, SD)** | | | | | | | | | | |
| --- | --- | --- | --- | --- | --- | --- | --- | --- | --- | --- |
|  | **Age (years)** | | **Gender (female)** | | **Education** | | **PSQI** | | **ISI** | |
|  | mean | SD | N | % | mean | SD | mean | SD | mean | SD |
| Completers  (N = 113) | 61.14 | 6.48 | 68 | 58.4% | 3.48 | 1.02 | 10.94 | 3.15 | 14.7 | 3.79 |
| Dropouts  (N = 14) | 59.29 | 5.58 | 6 | 28.6% | 4.08 | 1.04 | 10.86 | 2.57 | 13.7 | 5.61 |
| Group comparisons | t | p | X^a^ | p | U^b^ | p | t | p | t | p |
|  | -1.02 | 0.31 | 4.48 | 0.03 | 496 | 0.051 | -.92 | 0.93 | -0.62 | 0.55 |
| Abbreviations: SD, Standard Deviation; PSQI, Pittsburgh’s Sleep Quality Index; ISI, Insomnia Symptoms Index; p, p value of group comparison tests a. Chi t-test; b. Mann-Whitney U test. | | | | | | | | | | |
