# Supplementary material for: Mindfulness-based therapy for insomnia for older adults with sleep difficulties: a randomized clinical trial
Source: Psychol Med. 2021 Jul 1;53(3):1038–48. doi: 10.1017/S0033291721002476 (PMC9975962; doi:10.1017/S0033291721002476)
Supplement: Supplementary file 1 [file S0033291721002476sup001.zip › S0033291721002476sup008.docx]

Data Table 3 for Perini et al Mindfulness-Based Therapy for Insomnia for older adults with sleep difficulties: a randomized clinical trial

| **Table 3 \| PSG inter-rater reliability scoring on 72 PSG nights in total** | | | | | | |
| --- | --- | --- | --- | --- | --- | --- |
|  | **Rater 1** | | **Rater 2** | | **ICC** | **95% CI** |
|  | mean (mins) | SD (mins) | Mean (mins) | SD (mins) |  |  |
| SOL | 21.91 | 18.56 | 20.75 | 16.02 | 0.89 | 0.83 to 0.93 |
| WASO | 72.17 | 50.35 | 78.19 | 56.74 | 0.92 | 0.87 to 0.95 |
| TIB | 447.81 | 69.91 | 447.82 | 69.87 | 1 | 1.00 to 1.00 |
| TST | 353.67 | 67.71 | 348.88 | 73.01 | 0.95 | 0.93 to 0.97 |
|  | % | % | % | % |  |  |
| SE | 79.35 | 11.38 | 78.20 | 12.62 | 0.92 | 0.88 to 0.95 |
| Abbreviations: ICC, IntraClass Correlation; SD, Standard Deviation, CI, Confidence Interval, mins, minutes, SOL, Sleep Onset Latency; WASO, Wake After Sleep Onset; TIB, total Time in Bed; TST, Total Sleep Time; SE, Sleep Efficiency. | | | | | | |
