# Supplementary material for: Mindfulness-based therapy for insomnia for older adults with sleep difficulties: a randomized clinical trial
Source: Psychol Med. 2021 Jul 1;53(3):1038–48. doi: 10.1017/S0033291721002476 (PMC9975962; doi:10.1017/S0033291721002476)
Supplement: Supplementary file 1 [file S0033291721002476sup001.zip › S0033291721002476sup009.docx]

**Appendix B: Intervention syllabus for Mindfulness Based Therapy for Insomnia (B1) and Sleep Hygiene Education and Exercise programme (B2)**

**Appendix B1 Contents of the Mindfulness Based Therapy for Insomnia**

| **Description** | **Structure** |
| --- | --- |
| 1. Introduction and Overview  The first session starts with introductions by each group member, followed by an overview of the treatment program and of the principles of mindfulness meditation. Instructor then guides participants through mindful eating and sitting meditation. The model of sleep difficulties of the three P (Predisposing, Precipitating and Perpetuating factors) is then discussed, with focus on how Mindfulness may act on the perpetuating factors.  At the end of every session participants are given handouts with a summary of the session, a daily exercise log and audio guide to aid and track their personal practice. Participants are encouraged to practice at least once a day, 6 days a week for a minimum of 20 minutes.  Every week participants are also given some chapters from the book Full Catastrophe Living by Kabat-Zinn to read at home, optional. | 1. Introduction to mindfulness meditation 2. Mindful eating 3. Sitting meditation 4. Model of sleep difficulties   Home tasks: sleep diary, mindful eating (once a day), mindful breathing (once a day 15 minutes) |
| 2. Stepping Out of Autopilot  Session starts with 20 minutes sitting mediation, followed by a discussion on how to establish each participant’s meditation practice. Concepts like stepping out of automatic pilot and bringing awareness to the mind and body are explained. After a 30-minute body scan, teacher explains the importance of being aware and differentiate sleepiness vs tiredness, and how this can help with insomnia. Sleep hygiene is discussed as a way to notice any behavior that might be incompatible or may facilitate with sleep | 1. Body scan 2. Awareness and insomnia 3. Sleep hygiene   Home tasks: sleep diary, body scan meditation (once a day for 20 minutes); follow sleep hygiene instructions from now on |
| 3. Paying Attention to Sleepiness and Wakefulness  Session starts with mindful walking and body scan, followed by inquiry with the teacher, and discussion on how to reinforce participants’ meditation and mindful movements.  Behavioral instructions for stimulus control and sleep restriction are introduced and each participant was given an individualized program to follow. The program consists of a prescribed time in bed (TIBrx) based on the participant’s TIB calculated from the sleep diary of the previous weeks along with instructions to maintains a consistent wake time, not going to bed until sleepy, and getting out of bed if not falling asleep in 20 minutes, with a minimum TIBrx of 5.5 hours. Teacher explains about the importance of being aware of sleepiness and wakefulness, and how it relates to sleep consolidation.  During the remaining weeks, the latter part of the session is spent adjusting the TIBrx based on each participant’s progress and a discussion of any difficulties with the program encountered since the last session. | 1. Mindful walking 2. Sleep consolidation   Home tasks: sleep diary; alternate daily body scan and mindful walking (once a day for 30-45 minutes); follow sleep consolidation (fixed wake up time) |
| 4. Working with Sleepiness at Night  Session starts with mindful movements and sitting meditation, followed by a discussion on concerns and question on sleep consolidation. Teacher provides instructions on how to use stimulus control to work with sleeplessness, and how to reassociate the bed as a place where one sleeps.  Sleep consolidation is updated based on the calculated sleep efficiency of from the sleep diary of the previous week, increasing or decreasing the TIBrx, aiming for sleep efficiency between 80-85%. | 1. Mindful movement/mindful yoga 2. Sleep alteration 3. Stimulus control   Home tasks: sleep diary; alternate daily body scan and mindful movements (once a day for 30-45 minutes); follow adjusted sleep consolidation |
| 5. Territory of Insomnia  Teacher guides participants through mindful walking and body scan, followed by inquiry about the practice. The territory of insomnia, including the role of negative thoughts and psychophysiological factors, is then introduced and the model is discussed together. Participants discuss about the first half of the sleep program and their meditation practice done so far.  Sleep consolidation is reviewed and updated if necessary. | 1. Review of program (Halfway point) 2. Territory of insomnia (Psychophysiological insomnia)   Home tasks: sleep diary; alternate daily body scan, mindful movement and mindful breathing (once a day for 30-45 minutes); follow adjusted sleep consolidation |
| 6. Acceptance and letting go  Session begins with mindful movements and sitting meditation, and inquiry about practice. Teacher explains the importance of acceptance and letting go for working with thoughts and feelings in the territory of insomnia, then introduces the three minutes breathing space practice.  General discussion about the sleep program and practice is done. | 1. Acceptance and letting go 2. Three-minute breathing space 3. Review of progress   Home tasks: sleep diary if still needed for sleep consolidation; alternate daily body scan, mindful movement and mindful breathing (once a day for 30-45 minutes); 3-minute breathing; follow adjusted sleep consolidation |
| 7. Revisiting the Relationship with Sleep  Session begins with mindful movements and sitting meditation, and inquiry about practice. A discussion about participants’ relationship with sleep follows, then teacher leads participants through nurturing and depleting activities. General discussion about the sleep program and practice is done, and how informal practice can be including in everyday life. | 1. Relationship with sleep 2. Activity: Story on “Letting Go” 3. Activity: Nurturing/depleting exercise 4. Informal mindfulness meditation   Home tasks: same as previous week |
| 8. Eating Breathing and Sleeping Mindfulness  Session starts with mindful walking, sitting meditation and inquiry.  The final session consisted of a discussion and setting up of an action plan for future episodes of insomnia, and ways to continue mindfulness meditation beyond the program. | 1. Sitting meditation with choice-less awareness 2. Action plan for sleep difficulties 3. Closing ceremony   Home tasks: participants are encouraged to keep practicing daily |

**Audio guided meditations shared with participants for MBTI home practice**

- 20 minutes Breath Meditation by Steven Hickman <https://soundcloud.com/ucsdmindfulness/20-min-seated-meditation-by-steve-hickman>
- 20 minutes Body Scan by Steven Hickman <https://soundcloud.com/ucsdmindfulness/20-min-body-scan-by-steve-hickman>
- 35 minutes Mindful Movement by Deborah Rana <https://soundcloud.com/ucsdmindfulness/35-min-mindful-movement-by-deborah-rana>
- 45 minutes Body Scan by Steven Hickman <https://soundcloud.com/ucsdmindfulness/45-minute-body-scan-by-steve-hickman?in=ucsdmindfulness/sets/body-scan>
- 45 minutes Mindfulness of the Breath Practice by Steven Hickman <https://soundcloud.com/ucsdmindfulness/45-min-seated-meditation-by-steve-hickman>

**Appendix B2. Contents of the Sleep Hygiene Exercise and Education program**

| **Description** | **Structure** |
| --- | --- |
| 1. Introduction  The introduction to SHEEP lays the groundwork of sleep hygiene education and tailored exercises to be introduced week by week over the 8 sessions. This session covers the stages sleep, its difficulties, and the 3P model. The exercise introduced in this first session is the Diaphragmatic Breathing Technique. Participants are encouraged to do this practice throughout the day and are given an audio guide and printed instructions, and a daily exercise log to track their personal practice. | 1. Stages of sleep 2. Model of sleep difficulties 3. Diaphragmatic breathing   Home tasks: sleep diary; Diaphragmatic Breathing Technique (5-15 minutes) |
| 2. Sleep Hygiene and Environment  Participants are educated about principles of sleep hygiene and the appropriate environmental factors to consider for a good night’s sleep. The exercise introduced in the second session is the Progressive Muscle Relaxation. Participants are encouraged to do this practice any time they feel stressed or to add it to their pre-sleep routine. | 1. Sleep hygiene (Environment) 2. Room environment 3. Progressive Muscle Relaxation   Home tasks: sleep diary; Diaphragmatic Breathing Technique (5-15 minutes); Progressive muscle Relaxation (5-15 minutes) |
| 3. Sleep Hygiene and Lifestyle  The discussion continues around the topic of sleep hygiene and begins to introduce lifestyle changes. Participants discuss goal setting and the possible changes they could make to their daily routines. The exercise introduced is the Evening Exercise Routine. This routine was designed to target and relax muscle tension built up throughout the day. Participants were encouraged to add this to their pre-sleep routine. | 1. Stretching exercises 2. Sleep hygiene (Lifestyle)   Home tasks: sleep diary; Diaphragmatic Breathing Technique (5-15 minutes); Progressive muscle Relaxation (5-15 minutes); Evening exercise routine. |
| 4. Sleep Routine  The session starts with an open discussion about participants’ pre-sleep routine and habits. The exercise introduced in the fourth session is the Morning Exercise Routine. This routine includes a series of stretches to wake up the body and muscles to start the day. Participants were encouraged to add this to their morning routine over the next week. | 1. Morning stretching exercises 2. Pre-sleep routine   Home tasks: sleep diary; Diaphragmatic Breathing Technique (5-15 minutes); Progressive muscle Relaxation (5-15 minutes); Evening/Morning Exercise routine |
| 5. Impact of Sleep Difficulties  This is an educational session around the impact of sleep difficulties and poor sleep lifestyles on one’s health. Participants are introduced to creating SMART goals (Specific, Measurable, Achieving, Relevant, Time-bound) and begin to make a commitment to changes in their lifestyle. | 1. Impact of sleep difficulties 2. Setting SMART goals   Home tasks: as previous week + follow new sleep hygiene instructions |
| 6. Debunking Sleep Myths  The session aims to reframe participants’ beliefs and myths around lifestyle choices that affect sleep quality. Participants engage in a short cardio workout designed for the older adults by the Health Promotion Board (Singapore). | 1. Stretching exercises 2. Identifying and challenging sleep myths   Home tasks: as previous week+ stretching exercise |
| 7. Stress Management  Participants take part in a discussion to share current life stressors that may be affecting their sleep quality and an introduction to goal setting. Stress management strategies are covered by the instructor. | 1. Stress management   Home tasks: as previous week |
| 8. Moving Forward  The last session serves to wrap up all that was covered over the 8-weeks. This session gives the opportunity to clarify any outstanding doubts and questions regarding their sleep routines. The instructor also takes the participants through a commitment pledge to instill a sense of accountability and responsibility to moving forward with their new lifestyle changes to their sleep routines. | 1. Reviewing past sessions 2. Action plan for moving forward 3. Commitment pledge 4. Resources for use 5. Post-session feedback   Home tasks: participants are encouraged to keep practicing daily |

**Audio and video files shared with participants for SHEEP home practice**

- Progressive muscle relaxation (Supplemental audio file 1)
- Stretching exercise <https://www.healthhub.sg/programmes/71/healthy-ageing-exercise>
