# Supplementary material for: Mindfulness-based therapy for insomnia for older adults with sleep difficulties: a randomized clinical trial
Source: Psychol Med. 2021 Jul 1;53(3):1038–48. doi: 10.1017/S0033291721002476 (PMC9975962; doi:10.1017/S0033291721002476)
Supplement: Supplementary file 1 [file S0033291721002476sup001.zip › S0033291721002476sup011.pdf]

CLINICAL TRIAL PROTOCOL

PROTOCOL TITLE:
Improving sleep continuity through mindfulness training for better cognitive ageing

PROTOCOL NUMBER:

CIRB NUMBER: 2017/2830

PROTOCOL VERSION: Version 1.1
PROTOCOL DATE: 15/08/2020

PRINCIPAL INVESTIGATORS:
Julian Lim, Assistant Professor, National University of Singapore
Kinjal Doshi, Principal Clinical Psychologist, Singapore General Hospital

Table of Contents

1 BACKGROUND AND RATIONALE 6

1.1 GENERAL INTRODUCTION 6
1.2 RATIONALE AND JUSTIFICATION FOR THE STUDY 6
1.2.1 RATIONALE FOR THE STUDY PURPOSE 6
1.2.2 RATIONALE FOR DOSES SELECTED .... ERROR! BOOKMARK NOT DEFINED.
1.2.3 RATIONALE FOR STUDY POPULATION .... ERROR! BOOKMARK NOT DEFINED.
1.2.4 RATIONALE FOR STUDY DESIGN 6

2 HYPOTHESIS AND OBJECTIVES 7

2.1 HYPOTHESIS 7
2.2 PRIMARY OBJECTIVES 7
2.3 SECONDARY OBJECTIVES 7
2.4 POTENTIAL RISKS AND BENEFITS: 8
2.4.1 POTENTIAL RISKS 8
2.4.2 POTENTIAL BENEFITS 8

3 STUDY POPULATION 8

3.1 LIST THE NUMBER AND NATURE OF SUBJECTS TO BE ENROLLED 8
3.2 CRITERIA FOR RECRUITMENT AND RECRUITMENT PROCESS 8
3.3 INCLUSION CRITERIA 9
3.4 EXCLUSION CRITERIA 9
3.5 SUBJECT REPLACEMENT 9

4 STUDY DESIGN 9

4.1 RANDOMISATION AND BLINDING 9
4.2 CONTRACEPTION AND PREGNANCY TESTING 10
4.3 STUDY VISITS AND PROCEDURES 10
4.3.1 SCREENING VISITS AND PROCEDURES 11
4.3.2 STUDY VISITS AND PROCEDURES 12
4.3.3 FINAL STUDY VISIT: 14
4.3.4 POST STUDY FOLLOW UP AND PROCEDURES 14
4.4 DISCONTINUATION/WITHDRAWAL 15
4.4.1 DISCONTINUATION CRITERIA 15
4.4.2 DISCONTINUATION VISIT AND PROCEDURES 15

5 TRIAL MATERIALS 15

5.1 TRIAL PRODUCT (S) .... ERROR! BOOKMARK NOT DEFINED.
5.2 STORAGE AND DRUG ACCOUNTABILITY .... ERROR! BOOKMARK NOT DEFINED.

6 TREATMENT 15

6.1 RATIONALE FOR SELECTION OF DOSE .... ERROR! BOOKMARK NOT DEFINED.
6.2 STUDY DRUG FORMULATIONS .... ERROR! BOOKMARK NOT DEFINED.
6.3 STUDY DRUG ADMINISTRATION .... ERROR! BOOKMARK NOT DEFINED.
6.4 SPECIFIC RESTRICTIONS / REQUIREMENTS .... ERROR! BOOKMARK NOT DEFINED.
6.5 BLINDING .... ERROR! BOOKMARK NOT DEFINED.
6.6 CONCOMITANT THERAPY .... ERROR! BOOKMARK NOT DEFINED.

7 SAFETY MEASUREMENTS 15

7.1 DEFINITIONS 15
7.2 COLLECTING, RECORDING AND REPORTING OF ADVERSE EVENTS AND SERIOUS ADVERSE EVENTS TO CIRB 16

7.3 Collecting, Recording and Reporting of Serious Adverse Events (SAEs) to the Health Science Authority (HSA) 16
7.4 Safety Monitoring Plan 16
7.5 Complaint Handling 16

8 DATA ANALYSIS 16

8.1 Data Quality Assurance 16
8.2 Data Entry and Storage 17

9 SAMPLE SIZE AND STATISTICAL METHODS 17

9.1 Determination of Sample Size 17
9.2 Statistical and Analytical Plans 17

10 DIRECT ACCESS TO SOURCE DATA/DOCUMENTS 17

11 QUALITY CONTROL AND QUALITY ASSURANCE 18

12 ETHICAL CONSIDERATIONS 18

12.1 Informed Consent 18
12.2 Confidentiality of Data and Patient Records 18

13 PUBLICATIONS 18

14 RETENTION OF TRIAL DOCUMENTS 19

15 FUNDING AND INSURANCE 19

LIST OF ATTACHMENTS 20

PROTOCOL SIGNATURE PAGE

Protocol Title:
Improving sleep continuity through mindfulness training for better cognitive ageing

Protocol Number:

Protocol Version/ Date:
Version 1.1 15 August, 2020

Sponsor Name:
Julian Lim, Kinjal Doshi

Declaration of Investigator

I confirm that I have read the above-mentioned protocol and its attachments. I agree to conduct the described trial in compliance with all stipulations of the protocol, regulations and ICH E6 Guideline for Good Clinical Practice (GCP).

Principal Investigator Name: __ Kinjal Doshi ____________________

Principal Investigator Signature: ____________________

Date: 15 August 2020

PROTOCOL SIGNATURE PAGE

Protocol Title:
Improving sleep continuity through mindfulness training for better cognitive ageing

Protocol Number:

Protocol Version/ Date:
Version 1.1 15 August, 2020

Sponsor Name:
Julian Lim, Kinjal Doshi

Declaration of Investigator

I confirm that I have read the above-mentioned protocol and its attachments. I agree to conduct the described trial in compliance with all stipulations of the protocol, regulations and ICH E6 Guideline for Good Clinical Practice (GCP).

Principal Investigator Name: ___ Julian Lim ______________________________

Principal Investigator Signature: _____ 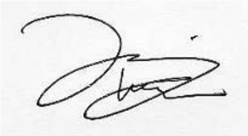 ____________________________

Date: _____15/08/20_____________________________________

1 BACKGROUND AND RATIONALE

1.1 General Introduction

Cognitive decline in the elderly is a rapidly growing concern in Singapore and other developed Asian nations. Among the major but understudied factors that may exacerbate this decline is poor sleep quality. Targeting sleep to decelerate or even reverse age-related cognitive decline may represent a low-cost, high-return solution to a widespread societal problem. To accomplish this goal, we propose to test mindfulness-based training (MBT) as an intervention to improve sleep quality and cognition.

1.2 Rationale and Justification for the Study

1.2.1 Rationale for the Study Purpose and Study Population

<u>Good sleep is critical for cognition at all ages, and especially in the elderly</u>

Adequate sleep is important for optimal cognitive function across the lifespan (Carskadon, 2011; Groeger et al., 2014; Lim & Dinges, 2010; Lo et al., 2012; O'Brien, 2009; Pace-Schott & Spencer, 2011). Although the association between sleep and cognitive function is bi-directional, alterations in sleep frequently occur prior to the appearance of cognitive symptoms in Alzheimer's Disease (AD) (Ju et al., 2014). Almost 50% of older adults report at least one sleep problem (Neikrug & Ancoli-Israel, 2010), and there is growing evidence that sleep complaints and disturbances might have negative effects on cognition (Yaffe et al., 2014).

Retired older adults have the freedom to sleep ad libitum but this benefit is offset by age-associated changes in sleep such as poorer subjective quality, increased fragmentation and/or altered macrostructure (Ohayon et al., 2004). Although sleep duration is the measure that has received the most attention (Lo et al., 2016), there is accumulating evidence that sleep quality (Blackwell et al., 2014; Nebes et al., 2009) and sleep fragmentation (Lim et al., 2013) in particular, can have deleterious effects on brain structure and cognition (Yaffe et al., 2014). A change in sleep pattern in late adulthood can foreshadow cognitive decline (Ferrie et al., 2011), perhaps as a result of accumulation of neurotoxic substances that are normally cleared during sleep (Ju et al., 2014).

<u>Mindfulness-based training improves sleep quality</u>

Mindfulness-based training (MBT) consists of a suite of techniques aimed at enhancing awareness and acceptance of one's internal (e.g., thoughts and feelings) and external experiences in the present moment (Kabat-Zinn, 1991). Recently, sleep experts have recognized the relevance of mindfulness-based interventions for treating the root causes of psychogenic sleep pathology. For example, one of the important causes of chronic primary insomnia is thought to be a failure to de-arouse. This could arise from maladaptive thoughts and beliefs about sleep. These may in turn be governed by metacognitive processes – "thoughts about thoughts" – a prime target for alteration by MBT (Ong et al., 2012; Teasdale, 1999).

Results from several small-scale randomized clinical trials of mindfulness-based interventions for primary insomnia have been reported (Gross et al., 2011; Ong et al., 2014; Zhang et al., 2015). These studies have generally shown positive results of the treatment with medium to large effect sizes, albeit on different outcome measures. The most consistently reported changes are seen in subjective measures of sleep quality (e.g. sleep diaries or questionnaires), but improvements have also been seen in total sleep and wake time as measured by actigraphy (Ong et al., 2014). Evidence for positive changes in sleep as assessed with polysomnography (PSG) is still scarce, as few studies to date have used this gold-standard measure (the most prominent study to date being Britton et al., 2012).

A recent randomized clinical trial demonstrated that MBT might also benefit those with moderate sleep disturbances but no clinical diagnosis. Black and colleagues (2015) recruited a cohort of 49 elderly

participants with self-reported poor-quality sleep, and found that mindfulness awareness practices were superior to sleep hygiene education in improving sleep quality, insomnia symptoms, depression symptoms, and fatigue severity. This last study suggests that mindfulness training might be a well-tolerated intervention that could be used to effectively counteract the decreases in sleep quality that are a natural consequence of ageing.

There is also evidence that mindfulness based stress reduction (MBSR) may be an effective treatment for sleep disturbances owing to other medical conditions (e.g. cancer). Andersen et al. (2013) studied the effects of an 8-week MBSR program on 336 women with Stage I to III breast cancer, and found short-term benefits for sleep quality. Garland et al. (2014) studied a cohort of patients with insomnia comorbid with cancer and found that MBSR was non-inferior to cognitive behavioral therapy in reducing insomnia symptoms. However, attrition was higher in the MBSR group, suggesting that acceptability of the treatment was poorer. Lengacher and colleagues (2015) studied a smaller group of breast cancer survivors (N = 79), and found significant beneficial effects of MBSR training on sleep efficiency and the number of waking episodes, as measured by actigraphy.

<u>Connecting the dots: mindfulness, sleep and cognition</u>

Poor or restricted sleep adversely affects many aspects of cognition, and one of its most pronounced and robust effects is on sustained attention (Lim & Dinges, 2010). This is important as attention is the bedrock of cognition: deficits in attention can have a domino effect, leading to problems in memory encoding, working memory, problem solving, and many other higher cognitive functions.

A distinct body of research has linked mindfulness training to improvement in multiple cognitive domains (Chisea et al., 2011), including sustained attention and executive attention. However, it is not yet known whether these effects are direct, or mediated by other factors such as sleep variables. To fill this knowledge gap, we propose to test the hypothesis that MBT will stabilize or improve cognition through the mediating effects of reduced sleep fragmentation and improved sleep quality.

1.2.2 Rationale for Study Design

We will use a block randomized controlled, parallel-group design to test the effects of mindfulness training on sleep and cognition in healthy elderly participants. This is the gold-standard design for testing the effects of a treatment on particular outcomes. Block randomization is necessary for this study because mindfulness classes are most effective when class size is relatively small (~10).

2 HYPOTHESIS AND OBJECTIVES

2.1 Hypothesis

1) Mindfulness-based training (MBT) will lead to greater improvements in subjective and objective sleep quality than sleep hygiene education (SHE).
2) MBT will lead to significantly greater improvements in sustained and executive attention relative to SHE (measured both behaviorally and using functional magnetic resonance imaging).
3) Cognitive outcomes will be mediated by improvements in subjective and objective sleep quality.

2.2 Primary Objectives

The objective of this protocol is to test whether MBT is superior to SHE in improving sleep quality (hypothesis #1) and cognition (hypothesis #2).

2.3 Secondary Objectives

The secondary objectives of the protocol are to assess the relative effects of MBT and SHE on structural and functional imaging methods, objective measurement of mindfulness (i.e. breath counting), pre-sleep arousal, depression, anxiety, and quality of life.

2.4 Potential Risks and Benefits:

2.4.1 Potential Risks

Risks due to Interventions
No major health risks and side effects are expected from participation. However, participants may become more aware of physical pain or experience some emotional distress due to participation in the programmes. To guard against health issues that may develop during the study, we will administer the 36-Item Short Form Survey at several points within the protocol. Participants who report low levels of physical and or mental QOL will be counseled by the PI who is a clinical psychologist, and if needed, referred for further counseling.

Risks due to fMRI procedures:
The risks of taking part in or conducting this study are minimal. Certain safety precautions have to be taken prior to MRI scanning (e.g. ensuring participants have no metal on their person). The Duke-NUS PI has conducted fMRI research for >10 years, and has a team that is fully trained in MR safety. There are no known long-term health risks to the use of magnetic resonance imaging per se when operated within standard (Food and Drug Administration, USA) guidelines. However, there are safety concerns posed by the strong magnetic fields used to make images. All scans conducted under this protocol will meet the FDA's guidelines for non-significant risk for static field strength, specific absorption rate (SAR), time varying magnetic fields (dB/dt), and acoustic noise. We will mitigate the risks associated with bringing ferromagnetic and other metallic objects into the scanner environment by strictly adhering to exclusion criteria, and through careful screening and wanding of subjects prior to scanning. Subjects may also feel dizzy or experience tingling sensations while in the magnet, or possible momentary loss of balance after leaving the magnet. These sensations are mostly due to movement while inside the magnet and can be minimized by staying still. All of these sensations should stop shortly after the subject leaves the magnet. Additionally, because of the small space in the magnet, and the duration of the study, some people can find the experiment to be uncomfortable or unpleasant. The investigator and the MR technician will check with the participant frequently to determine if they are having any such negative sensations.

Risks due to usage of Actigraphy:

The risks associated with this study are minimal and not more than those incurred through normal daily activities. The Actiwatch case is made of biocompatible ABS and the wristband of BASF Elastolian + titanium for the buckle. There are no known safety issues. However, you may experience some minor skin irritation from wearing the Actiwatch for prolonged lengths of time. We recommend that you dry the area beneath the watchband after showering or profuse perspiration. A small piece of soft cloth or tissue can also be inserted beneath the Actiwatch if there is any skin sensitivity.

2.4.2 Potential Benefits

Participants may reasonably expect to benefit from the Mindfulness-Based Training programme or the Sleep Hygiene Education programme. They may gain knowledge about the use of mindfulness interventions or effective techniques to deal with sleep problems. They can practice these techniques on their own to help manage their stress and address their sleep difficulties.

3 STUDY POPULATION

3.1 List The Number and Nature of Subjects to be Enrolled

120 participants will be recruited from the general public. There will be no exclusions based on race or sex.

3.2 Criteria for Recruitment and Recruitment Process

A phone and in-person screening will be used to determine study eligibility. Subjects will have to compete the Pittsburgh Sleep Quality Inventory, the Mini-Mental State Examination, and the Montreal Cognitive Assessment as part of this process. Participants will be recruited through advertising and word-of-mouth.

3.3 Inclusion Criteria

Inclusion Criteria:

a) Adults aged 50 – 80 years
b) English-speaking
c) Mini-Mental State Examination Score ≥ 26
d) Montreal Cognitive Assessment Score ≥ 23
e) At least one of the following sleep difficulties are expressed:
   (i) average reported sleep latency of more than 30 minutes
   (ii) average wakefulness after sleep onset of more than 30 minutes
   (iii) sleep efficiency or total sleep time of less than 6.5 hours
   and/or
   (iv) also a cut off in the Pittsburgh Sleep Quality Inventory >/=5

3.4 Exclusion Criteria

Exclusion Criteria:

a) Presence of major neurological conditions such as epilepsy, stroke, Parkinson's Disease and/or brain injury
b) Presence of major psychiatric conditions such as major depression or schizophrenia
c) Unsuitability for fMRI scanning (e.g. pacemakers, metallic implants, claustrophobia)
d) Unable to give independent consent or no consent available
e) Ongoing medication for sleep
f) Prior formal experience with mindfulness or meditation
g) Berlin Questionnaire ≥ 2 or use of continuous positive airway pressure device

3.5 Subject Replacement

Subjects who drop out will not be replaced.

4 STUDY DESIGN

4.1 Randomisation and Blinding

Participants are randomly assigned to interventions with 1:1 probability to be in either MBTI or SHEEP group. Randomization is performed only once all participants for one phase are enrolled in the study, and once both PSG and scan visits are completed, to avoid bias during the collection of pre-intervention data. In case it is necessary to assign participants to intervention before the last PSG/scan visit, due to time constrains, only blinded experimenters are allowed to perform the remaining visits. Participants are informed of their allocation only once they complete all visits pre-intervention. Before each phase randomization, it will be decided which experimenters will be unblinded and blinded, depending on the availability on assisting either during the intervention (unblinded team) or for testing the post intervention sessions (blinded team). Principal investigators of the project are also unblinded. Up to two additional assistants from the blind team will also be chosen to be unmasked in case of need, for example if the unblinded team needs additional help running the interventions.
For each phase of approximately 20 participants, a simple randomization method is computed using a customized script on Matlab. Randomization is run on the computer of the person in charge (i.e. Dr Francesca Perini), in the presence of a second person from the unblinded team as a witness. The output files are then password protected (only the unblinded team know the password). If one of the unblinded team will accidentally discover the assignment of one of the participants, they will not collect follow up measures for that participant, but can still assist for follow up visits for the remaining participants.

4.2 Contraception and Pregnancy Testing

N.A.

4.3 Study Visits and Procedures

Participant will be involved in the study for a total of 16 visits (see also Appendix 1).

Visit 1: Screening & Informed Consent. The study team member will administer the Mini-Mental State Examination (MMSE), Montreal Cognitive Assessment (MOCA) and the Pittsburg Sleep Quality Inventory (PSQI) to determine eligibility of the study. Participants who are found to not be eligible for the study will be withdrawn from the study. Participants who are found to be eligible will provide the study team with dates when they are available for the team to visit them at their home in the evening to set up the polysomnogram equipment and provide them with the actigraph watch.

Visit 2: 2 study team members will visit the participants' home in the evening approximately 2 hours before their bed-time. The study team members will introduce the participant to the polysomnogram equipment and set it up for them. The study team members will also introduce the participants to the use of the actigraph watch and help set it up for them. Participants will also complete base-line questionnaires related to sleep, mood, anxiety and quality of life.

Visit 3. One or two team members will visit the participant's home the next morning at a time convenient for the participant to collect the polysomnogram equipment and data collected with it.

Visit 4. Participant will be required to come to the Duke-NUS Cognitive Neuroscience Laboratory to return the actigraph watch to the study team. They will also undergo an MRI scan, which will take approximately 60 minutes. In addition, either before the MRI Scan or after, they will complete a set of computer-based cognitive tests and a set of cognitive tests administered using paper-and-pencil tasks conducted by a study team member.

Visit 5 - 12: Participant will attend either the mindfulness intervention program or the sleep hygiene program, which they were randomly assigned. Each weekly session for 8 weeks will lasts approximately 1.5 hours, and will be held either on Duke-NUS campus or SGH campus. The sessions will be led by study team members and instructors who have been trained in delivering the study intervention protocols. Participants will receive $10 a week to cover their travel expenses

Visit 13: 2 study team members will visit the participants' home in the evening approximately 2 hours before their bed-time. The study team members will familiarise the participant to the polysomnogram equipment and set it up for them again. The study team members will also familiarise the participants to the use of the actigraph watch and help set it up for them again. Participants will also complete base-line questionnaires related to sleep, mood, anxiety and quality of life.

Visit 14: One or two study team members will visit the participant's home the next morning at a time convenient for the participant to collect the polysomnogram equipment and data collected with it.

Visit 15: Participant will be required to come to the Duke-NUS Cognitive Neuroscience Laboratory to return the actigraph watch to the study team. They will also undergo an MRI scan, which will take approximately 60 minutes. In addition, either before the MRI Scan or after, they will complete a set of computer-based cognitive tests and a set of cognitive tests administered using paper-and-pencil tasks conducted by a study team member. Participants will receive $50 for their time and to cover their travel expenses. Participants will also complete base-line questionnaires related to sleep, mood, anxiety and quality of life.

Visit 16: Participants will be called by one of the study team members to answer follow-up questionnaires on their sleep, mood, anxiety and overall quality of life.

4.3.1 Screening Visits and Procedures

Eligible participants that present to Singapore General Hospital's Sleep Disorder Clinic, or interested participants from public talks and forums will be approached and recruited. Participants from the public may also be recruited through the distribution of flyers. These participants will be screened for eligibility into the study. Participants will be screened for baseline sleep and cognitive functional ability using the Mini-Mental State Examination, Montreal Cognitive Assessment and the Pittsburg Sleep Quality Inventory. Participants will also be screened for MRI suitability (e.g. presence of pacemakers, metal fragments in the body, and claustrophobia). Participants will be given a card to track all appointment visits.
Eligible participants will be provided all the necessary information regarding the study, including the potential benefits and risks associated with being involved in the study, and their responsibilities as a participant should they choose to consent to volunteer for the study. They will also be informed that it is voluntary, and they are free to withdraw from the study at any time with no consequences to the current medical care they receive at SGH.
The study team member who takes consent will also ask the eligible participant to summarize what they were told during the informed consent process in order to evaluate participant's capacity to understand what it means to volunteer as a participant for this study. Upon receiving consent, eligible participants will be asked to schedule their baseline visits for home based PSG (visit 2 and 3) and fMRI scan and cognitive tests (visit 4). There is no time limit between screening visit and next study visits, though interval should ideally be 1-60 days between visits visit 1 and visits 2,3 and 4.

Trained research assistants will administer the following questionnaires at Screening visit 1 to assess eligibility:

a. **Pittsburgh Sleep Quality Index, PSQI** (Buysse et al., 1989) is a 19-item self-rated questionnaire for evaluating subjective sleep quality over the previous month. The PSQI has a sensitivity of 89.6% and specificity of 86.5% for identifying cases with sleep disorder, using a cut-off score of 5.
b. **Mini Mental State Examination (MMSE) is** a 30-point questionnaire that is used extensively in clinical and research settings to measure cognitive impairment
c. **Montreal Cognitive Assessment (MOCA)** is a widely used screening assessment for detecting cognitive impairment. It was created in 1996 by Ziad Nasreddine in Montreal, Quebec. It was validated in the setting of mild cognitive impairment, and has subsequently been adopted in numerous other settings clinically.
d. **Beck's Depression Inventory, BDI-II** (Beck, Steer & Brown, 1996) is a 21- item questionnaire that evaluates the severity of depressive moodstates. Each item is rated between 0 (no symptom impact) and 3 (maximum symptom impact) with a maximum score of 63. Two domains are evaluated: the cognitive-affective and somatic domains. A higher total score indicates a greater severity of depression. The BDI-II has been validated in various populations and exhibits good psychometric properties.

In addition, if participant is eligible, the following questionnaires may be done either at screening visit 1 or at visit 4:

a. **State Trait Anxiety Inventory, STAI** (Julian, 2011) The STAI has 40 items, 20 items allocated to each of the S-Anxiety and T-Anxiety subscales. It is a self-report the presence and severity of current symptoms of anxiety and a generalized propensity to be anxious. Internal consistency alpha coefficients ranging from 0.86 to 0.95.
b. **Insomnia Severity Index (ISI)** evaluates an individual's level of tendency for insomnia on a 7-item questionnaire. Scores above 15 indicates moderate severity of clinical insomnia while scores above 22 are indicative of severe clinical insomnia. ISI internal consistency was excellent for population samples both in the community and clinical samples as well, (Cronbach α of 0.90 and 0.91 respectively).
c. **Pre-Sleep Arousal, PSA** (Nicassio et al., 1985) is a 16-item self report questionnaire having both cognitive and somatic dimensions of arousal. Scores on each subscale range from 8-40; high scores on both subscale indicate more arousal. The Cronbach alpha of this scale was .88 and test-retest reliability is .72.
d. **Berlin questionnaire, BQ (Netzer et al,. 1999)** reliably identifies middle-aged and older persons in the community who are at high-risk for OSA. It incorporates questions about snoring (category 1), daytime

somnolence (category 2), and hypertension and BMI (category 3). Patients are scored as being at *high-risk* for OSA if they had a positive score on two or more categories, while those who do not are scored as being at *low-risk.*
e. **Morningness- Eveningness Questionnaire, MEQ** (Horne and Ostberg, 1976) is a 19-item self-reported questionnaire to measure whether a person's circadian rhythm produces peak alertness in the morning, evening or in between.
f. **Dysfunctional Beliefs and Attitudes about Sleep, DBAS** (Morin, C. M., 1993) is a 30-item self reported questionnaire to measure people's beliefs and attitudes about their personal sleep situations. Items are ranked from 0, strongly disagree, to 10, strongly agree.
Participants will receive $10 to cover their travel expenses.

4.3.2 Study Visits and Procedures

Visits 2 and 3 Polysomnography (Visit 2 set-up in the evening, and 3 collection)

Visit 2 can be done at any point before the start of the intervention (visit 5), while visit 3 had to take place the day after visit 2.
Baseline data will be collected through objective sleep quality measures over 4 visits. 2 research assistants, to ensure the participant is comfortable the gender of the research assistants, will be matched to the participants' preference and also to ensure the safety of our staff.
They will conduct the set-up and collection of the polysomnography (PSG) at the convenience of the participant's home at his or her habitual bedtime. An actiwatch is also given to the participant, to wear for one week, and fill in a sleep diary for the same week.

a. **Wrist-worn actigraphy**
Wrist actigraphy is a convenient and reliable way to monitor sleep. It will be performed using research-grade, wrist-worn accelerometers that are purpose calibrated to detect sleep by inferring from movement patterns. Unlike consumer grade activity trackers (e.g. Fitbit, Jawbone, Garmin vivoactive), which are oriented to counting steps, floors climbed and calories burned, the systems used in this proposal (e.g. Philips Respironics AW-2) has been calibrated against polysomnography, the gold standard in sleep measurement.
They have been used in a multitude of research studies worldwide. In particular, actigraphy has been cross-validated against PSG in older women.
A button is provided for the volunteer to mark bedtime and rise time. The AW-2 used in the current study is waterproof to the IPX7 standard and can be worn throughout the day. Within the device is a photodiode that measures light. This provides information about participant light exposure that can be a useful adjunct to understanding sleep habits.
We will use actigraphy as an objective measure of sleep quality to be compared against the subjective measures obtained through the sleep questionnaires. Results from Actigraphy will be assessed in much the same way as the outcomes from the sleep questionnaires.

b. Home-based Polysomnography, PSG
PSG is a multi-parametric test used in the study of sleep and as a diagnostic tool in sleep medicine. PSG is a comprehensive recording of the bio-physiological changes that occur during sleep. It will be performed at night, at the convenience of the participants' home, The PSG monitors many body functions including brain (EEG), eye movements (EOG), muscle activity or skeletal muscle activation (EMG) and heart rhythm (ECG) during sleep.
(Note: Actiwatch could be passed to participants anytime between screening and intervention / within one month after intervention to capture sleep quality at baseline before/after intervention.)
Trained research assistants will administer the following questionnaires at Visit 2:
Short-Form 36, SF-36 is a 36-item self-report survey of health, including physical and mental health, with 8 scaled scores. Higher scale scores reflect better quality of health as measured by that scale.
Five Facet Mindfulness Questionnaire, FFMQ (Baer et et al., 2006) is a 39-item questionnaire developed as a reliable and valid comprehensive instrument to assess for five different aspects of mindfulness in a community: (observing, describing, acting with awareness, non-judging of inner experience, and non-reactivity to inner experience).
If there is not enough time to complete the questionnaires at visit 2, these can also be done at visit 4.

Visit 4: Cognitive Tests and scan

Visit 4 can be done at any point between screening visit 1 and start of the intervention (visit 5). Visit starts between 8:30 and 11:30, and last for approximately 6/7 hours.

Computer based cognitive assessment
a. **Cambridge Cognition** [http://www.cambridgecognition.com/] **CAM-COG,** This is a digital cognitive assessment tool for data collection. Key domains assessed in the CAM-COG test are on levels of Attention, Memory, Executive Function, Emotional and Social Cognition and Psychomotor Speed.
Pen and Pencil Tests
a. **IQ: Advanced Progressive Matrices I and II** This test is a nonverbal group test typically used in educational settings. It is a 60-item test used in measuring abstract reasoning and regarded as a non-verbal estimate of fluid intelligence.
b. **Processing Speed: Colour Trails A and B** (Strauss et al., 2006) Widely used instrument in neuropsychological assessment to measure for speed of cognitive processing and executive functioning.
The direct score of both parts A and B are represented by the time taken to complete each task.

fMRI Scan
A research assistant and a trained scanner operator will be present for all fMRI scans. Preparation time and the scan itself will last approximately 60mins.
a. **Resting State Scans,** will be conducted to determine the change in DMN/ECN connectivity and anti-correlation between DMN and task positive networks.
b. **Breath Counting Task**, is a behavioural measure of trait mindfulness found to be reliable and correlated with self-report mindfulness.
c. **Volumetric Scans**
Movie task or Simple Cognitive Task
d. **Meditation Task**
Letting Go Practice.

Participants will receive $40 for their time and to cover their travel expenses.

(VISIT 5 to 12: Interventions: (i) Mindfulness-Based Training (MBT) (ii)Sleep Hygiene Education (SHE)
Participants are randomised into one of two intervention groups a few days before start of the intervention by the unblended team.
For each phase of the interventions, 6-15 participants will be assigned to the MBT program and 6-15 participants to the SHE program.
Participants will have to attend the assigned program once a week for 8 weeks, and each session will last about two hours. Both programs will occur concurrently during the week (i.e. the MBT will begin and end on the same week as the SHE program). Both programs will be facilitated by trained personnel. Participants will be reimbursed SG$10 for their travel and time expenses for each week of Visit 4 to Visit 12. A total of SG$80 for full attendance of the program.

(i) Mindfulness-Based Training
Participants in the MBT program will meet weekly for 8 weeks (i.e. 2 months). The duration of each session will be about two hours. Each session will introduce a mindfulness exercise. Participants will be asked to focus their attention and develop awareness towards their internal states (i.e. bodily sensations, thoughts, and emotions) as well as external environment. The facilitator will guide the participants through each exercise, and encourage participants to share, discuss and learn from their experience of the exercise. The session will end with the participants evaluating and setting their goals for daily practice of the session's mindfulness exercise. To promote daily practice, participants will be provided with a manual that outlines the week's activities and logbook to record information about their experiences, including type of mindfulness exercise they chose for the day and the amount of time they dedicated to it.

(ii) Sleep Hygiene Education
Participants in the SHE program will meet weekly for 8 weeks (i.e. 2 months). The duration of each session will be about two hours. Each session will introduce a concept related to sleep and sleep hygiene. The

facilitator will provide the theory and rationale behind the concept, and encourage participants to share and discuss their experiences related to the concept. The session will end with the participants evaluating how to implement the specific concept in their daily lives, and its potential implications for their sleep. To promote implementation of these concepts, participants will be provided with a manual that outlines the concept and how they intend to apply it to their daily lives. Participants will be asked to record their experiences, and evaluate its use.

Sleep questionnaires (PSQI and PSAS) will be administered at week 4 of intervention.
A feedback Questionnaire will also be administered after the intervention at Week 8.

Visits 13 and 14 Polysomnography post intervention (Visit 13 set-up in the evening, and 14 collection)

Visit 13 should be done within 1 month from the end of intervention (visit 12), while visit 14 had to take place the day after visit 13.
Procedures carried on visit 13 and 14 are exactly the same as in visits 2 and 3.

Visit 15: Cognitive Tests and scan

Visit 15 should be done within 1 month from the end of intervention (visit 12). Visit starts between 8:30 and 11:30, and last for approximately 6/7 hours.
Procedures carried on visit 15 are the same as in visit 4, with the exception of the Ravens APM task.
At the completion of visits 13,14 and 15, participants will be reimbursed SG$ 50.

Visit 16: Six month follow up.
A 6 month follow-up Questionnaire will be administered 6 months after the last intervention session to follow-up with participants practice. Participants will be contacted on the phone to complete the following questionnaires:

- Pittsburgh Sleep Quality Index, PSQI
- Pre-Sleep Arousal, PSA
- Brief survey to check any ongoing practice of intervention exercises.

Notes:

1. The order of PSG visit (visit 2 and 3 / visit 13 and 14) and fMRI scan visit (visit 4 / visit 15) can be swapped if it's more convenient to schedule for participants.)
2. The study team will review both PSG and fMRI data after the respective study visits (visits 2,4,13 and 15). If data is not acceptable for analysis, participants will be given the option to redo PSG/fMRI if they are agreeable. There will be a reimbursement of $20 for the repeat fMRI visit.)

4.3.3 Final Study Visit:

The time from the screening procedures till completion of follow-up tests or examinations is approximately 6 months.
The last visit that participants are required to attend at Duke-NUS is visit 15 which should happen within 1 month from end of intervention. Visit 16 follow up is only conducted on the phone. From visit 15 (or visit 14 if this happens after visit 15) there are no special instructions given to the participants and they are free to return to their normal routine and sleep pattern.

4.3.4 Post Study Follow up and Procedures

There will be no post study follow up or procedures.

4.4 Discontinuation/Withdrawal

4.4.1 Discontinuation Criteria

Study recruitment will stop in the case of any severe adverse events for CIRB review.

The research study will be discontinued if more than half of the participants in either group encouraged to stop attending the program due to any physical, psychological or emotional distress they may experience from attending the program.

4.4.2 Discontinuation Visit and Procedures

Subjects may withdraw voluntarily from participation in the study at any time. Subjects may also withdraw voluntarily from receiving the study intervention for any reason. No further evaluations will be performed in either of these cases. If discontinuation occurs due to an adverse event, the participant will be provided with appropriate care under medical supervision until any symptoms resolve or the subjects' condition becomes stable.

5 TRIAL MATERIALS

N.A.

6 TREATMENT

N.A.

7 SAFETY MEASUREMENTS

7.1 Definitions

An adverse event (AE) is any untoward medical occurrence in a patient or clinical investigation subject administered a pharmaceutical product and which does not necessarily have a causal relationship with this treatment. An adverse event (AE) can therefore be any unfavourable and unintended sign (including an abnormal laboratory finding), symptom, or disease temporally associated with the use of a medicinal (investigational) product, whether or not related to the medicinal (investigational) product.

A serious adverse event (SAE) is any untoward medical occurrence that at any dose:

- results in death
- is life-threatening
- requires inpatient hospitalisation or prolongation of existing hospitalisation
- results in persistent or significant disability/incapacity, or
- is a congenital anomaly/birth defect

7.2 Collecting, Recording and Reporting of Adverse Events and Serious Adverse Events to CIRB

Reporting of adverse events involves the Principal Investigator submitting the SAE Reporting Form to CIRB within the stipulated timeframe. The Principal Investigator is responsible for informing the institution representative, the chairman medical board (when required by the institution for local SAE resulting in death), sponsor or regulatory bodies as required and appropriate.

Reporting timeline to CIRB:

- Local unexpected SAE resulting in death that are related events should be reported immediately - within 24 hours of the Principal Investigator becoming aware of the event. This should be followed by a full report within 7 calendar days.
- Local unexpected, life-threatening SAE that are related events should be reported as soon as possible but no later than 7 calendar days after the Principal Investigator is aware of the event. This should be followed by a full report within 8 additional calendar days.
- Local unexpected, not life-threatening SAE that are related events should be reported no later than 15 calendar days after the Principal Investigator is aware of the event.
- An increase in the rate of occurrence of Local expected SAE that are related events, which is judged to be clinically important, should be reported within 15 calendar days after the Principal Investigator is aware of the event.
- Local unexpected AE that are related events should be reported at least annually (together with Study Status Report for annual review).
- Non-local unexpected SAE that are fatal or life threatening and related should be reported not later than 30 calendar days after the Principal Investigator is aware of the event.

7.3 Collecting, Recording and Reporting of Serious Adverse Events (SAEs) to the Health Science Authority (HSA)

All SAEs that are unexpected and related to the study drug will be reported to HSA. Please refer to the HSA website for more information on Safety Reporting Requirements for Clinical Trials.

The investigator is responsible for informing HSA no later than 15 calendar days after first knowledge that the case qualifies for expedited reporting. Follow-up information will be actively sought and submitted as it becomes available. For fatal or life-threatening cases, HSA will be notified as soon as possible but no later than 7 calendar days after first knowledge that a case qualifies, followed by a complete report within 8 additional calendar days.

7.4 Safety Monitoring Plan

The Principal Investigators and Study Team Members will monitor and contact participants who are reporting significant physical discomfort and/ or mental distress. The investigator, a clinical psychologist, will meet with the participant to evaluate the severity of their distress, and provide the participant with appropriate referral to a counsellor, and if necessary, terminate the participant's involvement in the study.

7.5 Complaint Handling

Complaints will be addressed by the Principal Investigators or the Institutional Review Board.

8 DATA ANALYSIS

8.1 Data Quality Assurance

Participants will only complete data collection forms in person during the sessions in view of the study team members, and the data collection forms given to them will be labeled with the identifier unique to that

participant to ensure authenticity.
Sleep questionnaires and practice survey for 6 months follow up may also be conducted over the phone by a trained experimenter.

8.2 Data Entry and Storage

2 research team members will enter the data collected using the data collection forms into a database separately. These records will be reviewed against one another at the end of each data collection period to ensure that they are entered accurately and completely. The data will also be reviewed in its entirety by the PIs.

Research data will be kept in password-protected files, or under lock and key. Only Study Team Members have access to passwords/ key.

Research data will be coded with an identifier, with no participant identifying data stored. Data identifier will be stored in separate password-protected files. Only Study Team Members will have access to the password.

All hardcopy and electronic research data will be destroyed or deleted after six years as per SingHealth CIRB Guideline on Retention of Research Data and Records (Version 1.0 02 Jan 2014).

9 SAMPLE SIZE AND STATISTICAL METHODS

9.1 Determination of Sample Size

Based on prior literature, the effects of mindfulness training on subjective sleep variables are in the moderate (d = 0.46-0.61; from Gross et al., 2011) to large (d = 0.89; from Black et al., 2015) range. Using a relatively conservative estimate (d = 0.6) based on these results, and with the study powered at $\beta = 0.9$, we propose to recruit 120 participants (60 per study group) to this experiment.

9.2 Statistical and Analytical Plans

a. General Considerations

Primary variables have been registered with ClinicalTrials.gov (ID: NCT03677726). We will assess for a difference in change in these outcomes between groups (i.e. a significant interaction) using repeated-measures ANOVA, and change in individual groups using bootstrap analysis. Missing data will be filled in using multiple imputation.

b. Safety Analyses

As no major health risks and side effects are expected from participation, no Safety Analysis are planned to be conducted.

c. Interim Analyses

No interim analysis will be conducted.

10 DIRECT ACCESS TO SOURCE DATA/DOCUMENTS

The investigator(s)/institution(s) will permit study-related monitoring, audits and/or IRB review and regulatory inspection(s), providing direct access to source data/document.

11 QUALITY CONTROL AND QUALITY ASSURANCE

Data will be evaluated by the PIs after each study phase for accuracy and adherence with the protocol. Specifically, the PIs will ensure that the data are complete, and that the quality of the objective data (MRI and PSG) meets the standards expected in the scientific community.

12 ETHICAL CONSIDERATIONS

This study will be conducted in accordance with the ethical principles that have their origin in the Declaration of Helsinki and that are consistent with the Good Clinical Practice and the applicable regulatory requirements.

This final Clinical Trial Protocol, including the final version of the Participant Information Sheet and Consent Form, must be approved in writing by the Centralised Institutional Review Board (CIRB) and regulatory approval from Health Sciences Authority (HSA), prior to enrolment of any patient into the study.

The principle investigator is responsible for informing the CIRB and HSA of any amendments to the protocol or other study-related documents, as per local requirement.

12.1 Informed Consent

Eligible participants will be provided all the necessary information regarding the study, including the potential benefits and risks associated with being involved in the study, and their responsibilities as a participant should they choose to consent to volunteer for the study. The will also be informed that it is voluntary, and they are free to withdraw from the study at any time with no consequences to the current medical care they receive at SGH. The study team member who takes consent will also ask the eligible participant to summarize what they were told during the informed consent process in order to evaluate participant's capacity to understand what it means to volunteer as a participant for this study.

Study team members who are eligible to take consent are specified on the CIRB application for this protocol. We are not recruiting subjects who are non-English speakers, children, illiterate, non-writing, or vulnerable populations.

A study participation form and MRI screening form are needed as part of consent for this study.

12.2 Confidentiality of Data and Patient Records

Sections 8 and 14 cover the measures put in place to ensure patient confidentiality.

13 PUBLICATIONS

The study team will communicate the results of this study in peer-reviewed journals and academic conferences. Study team members will be granted co-authorship if they have contributed substantively to one of the following: conceptualizing the study, analysing and interpreting the data, preparing and critically revising the manuscript. Study team members will be acknowledged if they contributed to the study in any substantive way other than what is listed above (e.g. data collection and management). The PIs of the study will have the final decision on the order of the author list, and the journal that manuscripts are submitted to.

14 RETENTION OF TRIAL DOCUMENTS

All hardcopies of research data will be kept under lock and key in the PI's Office in SGH and Duke-NUS. Electronic data exported for analysis will be de-identified, password-protected, encrypted and stored on a stand-alone PC that can only be assessed using unique user ID issued by the hospital. IRB records and other regulatory documentation will be retained on PI workstations with password protection.

All hardcopy and electronic research data will be destroyed or deleted after six years as per SingHealth CIRB Guideline on Retention of Research Data and Records (Version 1.0 02 Jan 2014).

15 FUNDING and INSURANCE

Funding for this study was provided by the Singapore Millennium Foundation, the Far East Organization, and a Duke-NUS start-up grant to Dr. Lim. Clinical trial insurance is provided by Duke-NUS Medical School.

List of Attachments

Appendix 1 Study Schedule

Appendix 2 List of References

Appendix 1: Study Schedule

Procedures	Visits															
	1	2	3	4	5	6	7	8	9	10	11	12	13	14	15	16
Screening & Informed Consent	X															
Objective Assessment of Sleep (PSG)		X	X										X	X		
Subjective Assessment of Sleep Questionnaires	X	X		X				X					X		X	
Mood and Anxiety Questionnaires		X		X									X		X	
fMRI Scan				X											X	
Cognitive Tests Computer- Based and Pen and Paper Tasks				X											X	
Mindfulness Based Training (MBT)					X	X	X	X	X	X	X	X				
Sleep Hygiene Education (SHE)					X	X	X	X	X	X	X	X				
Qualitative Feedback on Sessions												X				
6 Months Follow- Up Questionnaires																X
Time/ Duration (hours)	**1h**	**1.5h**	**0.5h**	**1h**	**2 h**	**2 h**	**2 h**	**2 h**	**2 h**	**2 h**	**2 h**	**2 h**	**1.5h**	**0.5h**	**1h**	**0.5h**
Reimbursements	**$10**			$40	$10	$10	$10	$10	$10	$10	$10	$10			$50	

(Note: The order of PSG visit (visit 2 and 3) and fMRI scan visit (visit 4), or visits 13/14 and visit 15, can be swapped if it's more convenient to schedule for participants.).

Appendix 2: List of References

Blackwell, T., Yaffe, K., Laffan, A., Ancoli-Israel, S., Redline, S., Ensrud, K. E., et al. (2014). Associations of objectively and subjectively measured sleep quality with subsequent cognitive decline in older community-dwelling men: the MrOS Sleep Study. Sleep, 37, 655-663.

Ferrie, J. E., Shipley, M. J., Akbaraly, T. N., Marmot, M. G., Kivimaki, M., & Singh-Manoux, A. (2011). Change in sleep duration and cognitive function: findings from the Whitehall II Study. Sleep, 34, 565- 573.

Lim, A. S., Kowgier, M., Yu, L., Buchman, A. S., & Bennett, D. A. (2013). Sleep fragmentation and the risk of incident Alzheimer's Disease and cognitive decline in older persons. Sleep, 36, 1027-1032.

Nebes, R. D., Buysse, D. J., Halligan, E. M., Houck, P. R., & Monk, T. H. (2009). Self-reported sleep quality predicts poor cognitive performance in healthy older adults. J Gerontol B Psychol Sci Soc Sci, 64, 180-187.

Ohayon, M. M., Carskadon, M. A., Guilleminault, C., & Vitiello, M. V. (2004). Meta-analysis of quantitative sleep parameters from childhood to old age in healthy individuals: developing normative sleep values across the human lifespan. Sleep, 27, 1255-1273.
