# Supplementary material for: Mindfulness-based therapy for insomnia for older adults with sleep difficulties: a randomized clinical trial
Source: Psychol Med. 2021 Jul 1;53(3):1038–48. doi: 10.1017/S0033291721002476 (PMC9975962; doi:10.1017/S0033291721002476)
Supplement: Supplementary file 1 [file S0033291721002476sup001.zip › S0033291721002476sup012.docx]

Additional ITT rmANOVA for Perini et al Mindfulness-Based Therapy for Insomnia for older adults with sleep difficulties: a randomized clinical trial

| **Table 4. Repeated Measures ANOVA F values for ITT analysis using gender as covariate** | | | | | | | | | | | | | | | | | |
| --- | --- | --- | --- | --- | --- | --- | --- | --- | --- | --- | --- | --- | --- | --- | --- | --- | --- |
|  | **Primary Measures** | | | | | | **Secondary Measures** | | | | | | | | | | |
|  | **PSQI** | **ISI** | **PSG** | | **Actigraphy** | | **FFMQ** | **PSAS** | | **DBAS** | **PSG** | | | **Actigraphy** | | |  |
|  |  |  | WASO | SOL | WASO | SOL |  | Somatic | Cognitive |  | TST | TIB | SE | TST | TIB | SE |  |
| **ITT Analysis** | | | | | | | | | | | | | | | | | |
| Time | 22.09^a^ | 18.31^a^ | 1.75 | 0.95 | 0.06 | 0.12 | 1.62 | 1.01 | 4.41^b^ | 5.73^b^ | 2.38 | 0.87 | 1.02 | 0.35 | 0.08 | 0.54 |  |
| Time*Gender | 1.50 | 0.78 | 0.34 | 0.81 | 0.98 | 1.06 | 0.54 | 0.22 | 0.31 | 0.02 | 0.06 | 0.14 | 0.13 | 0.09 | 0.53 | 1.75 |  |
| Time*Group | 0.40 | 7.12^a^ | 0.80 | 0.08 | 5.40^b^ | 0.92 | 0.77 | 0.09 | 0.23 | 1.62 | 0.21 | 0.03 | 0.46 | 0.04 | 2.31 | 2.21 |  |
| Abbreviations: MBTI, Mindfulness Based Therapy for Insomnia; SHEEP, Sleep Hygiene Exercise and Education program; SD, Standard Deviation; PSQI, Pittsburg’s Sleep Quality Index; ISI, Insomnia Symptoms Index; WASO, Wake After Sleep Onset; PSG, Polysomnography; SOL, Sleep Onset Latency; FFMQ, Five Facets Mindfulness Questionnaire; PSAS, Pre Sleep Arousal Scale; DBAS, Dysfunctional Beliefs about Sleep; TST, Total Sleep Time; TIB, total Time in Bed; SE, Sleep Efficiency. a = p value <0.01; b = p value <0.05; | | | | | | | | | | | | | | | | | |
