# Supplementary material for: Mindfulness-based therapy for insomnia for older adults with sleep difficulties: a randomized clinical trial
Source: Psychol Med. 2021 Jul 1;53(3):1038–48. doi: 10.1017/S0033291721002476 (PMC9975962; doi:10.1017/S0033291721002476)
Supplement: Supplementary file 1 [file S0033291721002476sup001.zip › S0033291721002476sup013.docx]

Additional ITT rmANOVA for Perini et al Mindfulness-Based Therapy for Insomnia for older adults with sleep difficulties: a randomized clinical trial

| **Table 5. Repeated Measures ANOVA F values for ITT analysis using BDI and/or STAI as predictors during imputation** | | | | | | | | | | | | | | | | | |
| --- | --- | --- | --- | --- | --- | --- | --- | --- | --- | --- | --- | --- | --- | --- | --- | --- | --- |
|  | **Primary Measures** | | | | | | **Secondary Measures** | | | | | | | | | | |
|  | **PSQI** | **ISI** | **PSG** | | **Actigraphy** | | **FFMQ** | **PSAS** | | **DBAS** | **PSG** | | | **Actigraphy** | | |  |
|  |  |  | WASO | SOL | WASO | SOL |  | Somatic | Cognitive |  | TST | TIB | SE | TST | TIB | SE |  |
| **ITT Analysis (STAI only as predictor in imputation): main analysis in paper** | | | | | | | | | | | | | | | | | |
| Time | 118.57^a^ | 113.12^a^ | 5.70^b^ | 0.15 | 4.53^b^ | 3.71 | 3.18 | 2.99 | 23.69^a^ | 60.93^a^ | 16.28^a^ | 3.27 | 17.43^a^ | 0.95 | 8.92^a^ | 2.49 |  |
| Time*Group | 0.31 | 6.89^a^ | 0.75 | 0.12 | 5.68^a^ | 1.04 | 0.71 | 0.08 | 0.21 | 1.66 | 0.23 | 0.03 | 0.44 | 0.05 | 2.46 | 2.43 |  |
| **ITT Analysis (BDI only as predictor in imputation)** | | | | | | | | | | | | | | | | | |
| Time | 114.76^a^ | 128.23^a^ | 8.66^a^ | 0.08 | 4.43^b^ | 3.48 | 5.74^b^ | 3.22 | 24.10^a^ | 58.00^a^ | 8.19^a^ | 0.02 | 20.01^a^ | 0.94 | 8.51^a^ | 2.85 |  |
| Time*Group | 0.91 | 9.29^a^ | 0.33 | 0.82 | 5.99^b^ | 1.59 | 2.54 | 0.05 | 0.30 | 1.41 | 0.14 | 0.18 | 0.48 | 0.06 | 2.75 | 2.85 |  |
| **ITT Analysis (both BDI and STAI as predictors in imputation)** | | | | | | | | | | | | | | | | | |
| Time | 121.85^a^ | 125.45^a^ | 7.25^a^ | 0.03 | 4.17^b^ | 3.40 | 7.44^a^ | 2.97 | 23.71^a^ | 69.16^a^ | 7.46^a^ | 0.01 | 15.83^a^ | 0.97 | 8.59^a^ | 2.06 |  |
| Time*Group | 0.77 | 7.82^a^ | 1.57 | 0.21 | 6.59^a^ | 0.99 | 3.23 | 0.16 | 0.17 | 1.99 | 0.07 | 0.05 | 0.14 | 0.08 | 2.49 | 2.62 |  |
| Abbreviations: BDI, Beck’s Depression Inventory; STAI, State/Trait Anxiety Inventory; MBTI, Mindfulness Based Therapy for Insomnia; SHEEP, Sleep Hygiene Exercise and Education program; SD, Standard Deviation; PSQI, Pittsburg’s Sleep Quality Index; ISI, Insomnia Symptoms Index; WASO, Wake After Sleep Onset; PSG, Polysomnography; SOL, Sleep Onset Latency; FFMQ, Five Facets Mindfulness Questionnaire; PSAS, Pre Sleep Arousal Scale; DBAS, Dysfunctional Beliefs about Sleep; TST, Total Sleep Time; TIB, total Time in Bed; SE, Sleep Efficiency. a = p value <0.01; b = p value <0.05; | | | | | | | | | | | | | | | | | |
